# A CPG‐Based Versatile Control Framework for Metameric Earthworm‐Like Robotic Locomotion

**DOI:** 10.1002/advs.202206336

**Published:** 2023-02-12

**Authors:** Qinyan Zhou, Jian Xu, Hongbin Fang

**Affiliations:** ^1^ Institute of AI and Robotics State Key Laboratory of Medical Neurobiology MOE Engineering Research Center of AI & Robotics Fudan University Shanghai 200433 China

**Keywords:** bioinspired robot, central pattern generators, gait generation, gait transition, spatiotemporal dynamics

## Abstract

Annelids such as earthworms are considered to have central pattern generators (CPGs) that generate rhythms in neural circuits to coordinate the deformation of body segments for effective locomotion. At present, the states of earthworm‐like robot segments are often assigned holistically and artificially by mimicking the earthworms’ retrograde peristalsis wave, which is unable to adapt their gaits for variable environments and tasks. This motivates the authors to extend the bioinspired research from morphology to neurobiology by mimicking the CPG to build a versatile framework for spontaneous motion control. Here, the spatiotemporal dynamics is exploited from the coupled Hopf oscillators to not only unify the two existing gait generators for restoring temporal‐symmetric phase‐coordinated gaits and discrete gaits but also generate novel temporal‐asymmetric phase‐coordinated gaits. Theoretical and experimental tests consistently confirm that the introduction of temporal asymmetry improves the robot's locomotion performance. The CPG‐based controller also enables seamless online switching of locomotion gaits to avoid abrupt changes, sharp stops, and starts, thus improving the robot's adaptability in variable working scenarios.

## Graphical abstract

This work pioneers neurobiologically inspired control in metameric worm‐like robots. A generic, biologically plausible, versatile, and strongly adaptable control framework for metameric earthworm‐like robots is established to achieve polymorphism and better performance in metameric robotic locomotion. Seamless and smooth online switching of earthworm‐like robotic locomotion gaits is also realized to avoid abrupt changes and sharp stops or starts.

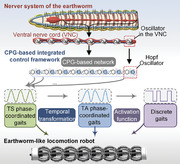

## 1 Introduction

Controller effectiveness and flexibility are critical factors for bioinspired limbless locomotion robots to acquire excellent mobility in unstructured environments.^[^ ^1^, ^2^, ^3^ ^]^ Based on morphological biomimicry, two conventional controllers, namely the discrete gait controller^[^ ^4^, ^5^, ^6^ ^]^ and the phase coordination controller,^[^ ^7^, ^8^, ^9^ ^]^ have enabled bio‐inspired metameric robots to effectively perform worm‐like and snake‐like locomotion in structured environments by intuitively mimicking the locomotory waves of limbless species. However, adapting to real‐world environments and variable tasks presents significant technological challenges to the above control strategies since the states of all robot modules corresponding to different motion patterns have to be calculated holistically and specified a priori, which is tedious, time consuming, and highly user‐dependent. This, therefore, calls for the extension of robotic biomimicry from the morphological level to the neuroethological level by mimicking the nervous system to explore a neuro‐inspired control approach for robotic locomotion, which requires the interdisciplinary integration of neurobiology and dynamics.

Neurobiologists have suggested that basic rhythmic activities, such as repetitive locomotion and cyclic respiration, are automatically regulated by self‐contained neural circuits, which are called central pattern generators (CPGs), without the involvement of higher regions in the brain.^[^ ^10^, ^11^ ^]^ Fundamentally, the CPG is a series of distributed oscillators located in the spinal cord of some vertebrates, such as rats,^[^ ^12^ ^]^ turtles,^[^ ^13^ ^]^ salamanders,^[^ ^14^ ^]^ and zebrafish.^[^ ^15^ ^]^ The CPG can generate complex rhythmic patterns of motor behavior without sensory input or feedback and can respond effectively to changes through proper activation of the descending neurons.^[^ ^16^, ^17^ ^]^ In particular, CPG, as part of the neural circuitry, can be modulated to adapt to the needs and the surroundings of the organism.^[^ ^18^ ^]^ For example, neuromodulators can change the intrinsic properties and the synaptic strength of neurons, which can alter the output patterns of the CPG circuit and thereby generate different motor patterns.^[^ ^19^, ^20^ ^]^

Previous works in robotics have elucidated that in CPG‐based control, the synchronized and phase‐related outputs of the coupled oscillators provide an efficient way of generating complex robot motion patterns; by modulating the activity of the CPG, the states of robot modules at each step can be specified locally by the CPG itself, which greatly reduces the dimensionality of the control inputs;^[^ ^16^, ^21^ ^]^ the CPG can also switch the locomotion gaits smoothly and continuously to adapt to different environments and tasks, thus improving the versatility of the robot in multiple scenarios.^[^ ^22^, ^23^ ^]^ Due to these distinct merits, as a successful practice of neuroscience in robotics, CPG‐based distributed control has become a highly influential concept in robotics research, partially replacing the model‐based control mechanism.^[^ ^24^, ^25^ ^]^

The CPGs play a similarly critical role in coordinating invertebrate behavior.^[^ ^26^, ^27^, ^28^ ^]^ The earthworm locomotion is also produced in part due to a series of distributed oscillators (i.e., the CPG) that are located within the segmental ganglion of the ventral nerve cord (VNC).^[^ ^29^, ^30^ ^]^ In this study, by synthesizing neuroscience with robotic technology, we present for the first time a versatile CPG‐based decentralized control framework for metameric earthworm‐like robots that can generate flexible rectilinear peristaltic locomotion.

Specifically, our work establishes an unorthodox paradigm for neuromorphic control architecture in which the distributed oscillators serve as a mapping between neural oscillations and robot segment deformations, and further exploits the collective dynamics of the oscillator network to generate complex patterns for locomotion control of the robot. Similar to biological CPG networks, in which the neuromodulators can work individually or jointly to alter the functional configuration of the network and thus adjust the output patterns,^[^ ^31^ ^]^ the constructed bioplausible CPG circuit of the earthworm‐like robot can effectively transform the global spatiotemporal dynamics so that the waveform and the phase difference are continuously adjustable, and it can also map continuous dynamics to discrete dynamics through activation functions. As a consequence, analogous to a polymorphic network, the CPG‐based control framework, through individual or joint activation of different modulators, can not only reproduce the existing phase‐coordinated gaits and discrete gaits but also generate new asymmetric wave control to acquire higher average locomotion speeds than the state‐of‐the‐art. The CPG‐based controller possesses the unique merit that it can spontaneously generate the actuation signals for all robot segments, which is completely different from the brain‐based control, where the states of all robot segments at each time step need to be artificially specified. This advantage is of particular interest when the number of robot cells increases dramatically. In addition, by harnessing the transit dynamics when evolving between fixed points and stable limit cycles or between different stable limit cycles, smooth and continuous transitions for starting, braking, and gait switching of the earthworm‐like robot can be achieved. Using a metameric earthworm‐like robot prototype (with the constitutive module driven by a muscle‐like antagonistic structure) as a testbed, rectilinear locomotion with a rich set of gaits is implemented in the proposed control framework, demonstrating the effectiveness, flexibility, and versatility of the CPG‐based approach. The contribution of this work provides a novel and robust building concept for achieving bioinspired control, beyond bioinspired structures, in metameric worm‐like robots, thus laying a solid foundation for attaining autonomy and adaptivity of peristaltic locomotion.

## 2 Results

### 2.1 CPG‐Based Network

**Figure 1:**
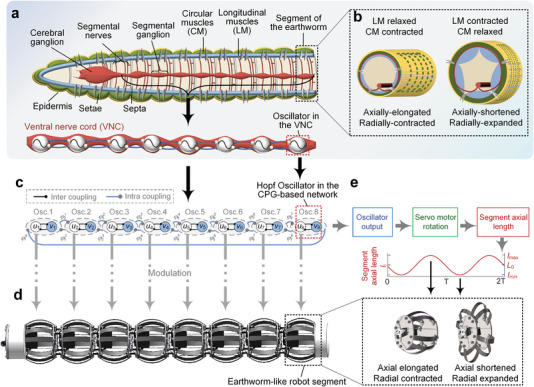
The CPG‐based network inspired by the nervous system of the earthworm. a) Schematic illustration of the earthworm's longitudinal section and the ventral nerve cord (VNC). b) A sketch of the earthworm's body segment in two states. c) The CPG‐based network consists of coupled Hopf oscillators with a topology inspired by the earthworm's nervous system. d) The eight‐segment earthworm‐like robot is controlled by the modulated outputs of the CPG‐based network. e) The output of the Hopf oscillator *u_i_* is converted to servo motor rotation angle for controlling the axial length *l_i_* of the robot segment, which as a consequence, exhibit antagonistic axial and radial deformations similar to the earthworm's body segment.

The design of the CPG‐based network is neurobiologically inspired by the well‐developed segmented nervous system of the earthworm (**Figure** **1**a). A ganglionic swelling of the VNC is found in each segment,^[^ ^32^, ^33^ ^]^ where distributed oscillators, perceived as the CPG, are present.^[^ ^29^, ^30^ ^]^ With the rapid conduction of nerve impulses through the giant fibers,^[^ ^34^ ^]^ it is likely that the oscillators locally provide segmental motion information to the radial and longitudinal muscles via segmental nerves extending from the ganglion, thus allowing the body segment to alternate between the axially elongated (radially contracted) state and the axially shortened (radially expanded) state^[^ ^33^ ^]^ (Figure 1b). Without the involvement of the cerebral ganglia,^[^ ^32^ ^]^ neurons in the CPG generate rhythmic patterns to control the periodic deformations of each segment, resulting in a self‐coordinated and self‐synchronized activity of the segmented body. Inspired by the abovementioned neural system and neural behavior of the earthworm, a CPG‐based network consisting of coupled oscillators (Figure 1c) is established for the earthworm‐like robot (Figure 1d, detailed in Experimental Section) to map the underlying dynamics of the nervous system to the locomotion of the robot. The CPG‐based network creates appropriate phase differences for the robot segments to achieve peristaltic locomotion through commensurate coupling between oscillators. Both the intrinsic properties of the oscillators and their connections are crucial for rhythm generation.

Specifically, the Hopf oscillator (see details in Experimental Section) is chosen as the mathematical implementation of the constituent unit of the CPG‐inspired network due to its structural simplicity, fast convergence rate, and excellent robustness to disturbances, as well as its well‐defined, mutually independent, and flexibly adjustable parameters.^[^ ^35^, ^36^, ^37^ ^]^ These merits are particularly advantageous when dealing with large and complex systems such as the multisegment earthworm‐like robot in this work. The Hopf oscillator can output a stable limit cycle on the *u* − *v* phase portrait (see discussions in Note S1 and Figure S1a, Supporting Information) due to the Hopf bifurcation of the dynamical system, manifested as the destabilization of the fixed point and the emergence of a stable periodic solution. Regardless of initial conditions and external perturbations, the stable limit cycle could guarantee the convergence of nearby trajectories, which is crucial for the robot working in unstructured environments to avoid unexpected interruptions of the stable continuous motion.^[^ ^38^, ^39^ ^]^ The outputs *u* and *v* are presented as periodic sine waves with a phase difference of *π*/2 in the time domain (see Figure S1b, Supporting Information), and they have the same amplitude and frequency to produce coordinated deformations of the circular and longitudinal “muscles” of the earthworm‐like robotic segment. Considering that the antagonistic deformations of the circular and longitudinal “muscles” in the robot^[^ ^40^ ^]^ are physically locked (see details in Experimental Section), only the rhythmic signal *u* for axial deformations is needed for each robot segment to simultaneously execute radial deformations and state switches (Figure 1e), similar to the earthworm's body segment (Figure 1b).

Rhythmicity is meaningless for an isolated neuron, whereas in coupled neurons, complex activities can arise under the synchronization and evolution of phase patterns. Hence, the next focus is on the neurobiologically inspired connectivity in the CPG‐based network and the intriguing spatiotemporal dynamics of the network. Mimicking the segmentally VNC that runs through the earthworm body,^[^ ^33^, ^41^ ^]^ oscillators that correspond one‐to‐one with the earthworm‐like robot segments are constructed as a chain configuration (Figure 1c). Given that both the anteroposterior and posteroanterior conduction of impulses is found in the giant fibers of VNC,^[^ ^42^ ^]^ the bidirectional coupling is set up between neighboring oscillators. We further find that an open‐loop chain structure brings free but inaccurate phase patterns, while a bidirectional coupling ring structure firmly constrains the sum of the phase differences of all neighboring oscillators to a multiple of 2*π*, and only a unidirectional coupling of the tail and head oscillators can maintain reliable and flexible dynamics of the network.

Figure 1c displays the topology structure of the CPG‐based network, where $\varphi_{i+1}^{i}=-\varphi_{i}^{i+1}=\varphi_{i}-\varphi_{i+1}$ denotes the phase difference of the *i* ^‐th^ oscillator to the (*i* + 1)^‐th^ oscillator for *i* = 1, …, *N* − 1, and without loss of generality, $\varphi_{i+1}^{i}\in \left[-\pi ,\pi \right]$. The phase difference for the unidirectional coupling from the tail oscillator to the head oscillator is $\varphi_{1}^{N}=2\pi -\sum_{i=1,\text{…}N-1}\varphi_{i+1}^{i}$. Note that starting from an unsynchronized initial condition, the outputs of the oscillators can be synchronized rapidly according to the speed and strength determined by the parameters and can remain phase‐locked in the steady‐state pattern determined by $\varphi_{i+1}^{i}$ (Figure S1, Supporting Information). Such a CPG‐based network can serve as a basis for constructing decentralized controllers for metameric earthworm‐like robots because of its capability in spontaneously generating phase‐locked and fast‐convergent spatiotemporal dynamic signals.

### 2.2 CPG‐Based Versatile Control Framework

**Figure 2:**
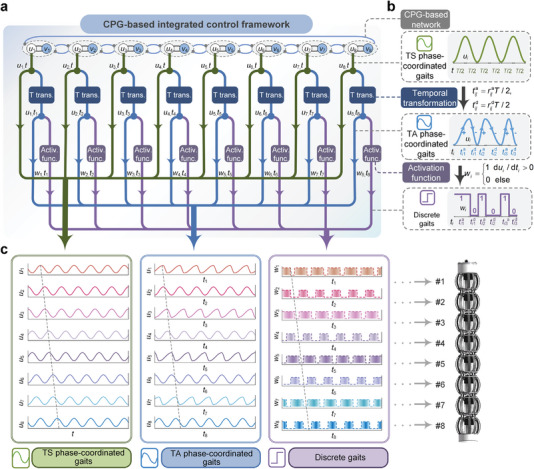
The CPG‐based versatile control framework. a) The architecture of the control framework consisting of the CPG‐based network, the temporal transformation module, and the activation function module. b) Evolution of the signal that sequentially undergoes the CPG‐based network, temporal transformation, and activation function. c) Examples of the TS and TA phase‐coordinated gaits and the discrete gait G9; their parameters are provided in Table S1, Supporting Information.

The biological CPGs are known as “polymorphic networks”^[^ ^43^, ^44^ ^]^ that can produce different motions applicable to specific tasks by activating or inhibiting neuromodulators. Learning from and applying it to robotics, the spatiotemporal dynamic properties of gaits should be adjustable in the CPG‐based network to adapt to different environments and tasks. To this end, functional components are integrated with the proposed CPG‐based network to construct a versatile control framework (**Figure** **2**a,b) to produce qualitatively different locomotion gaits.

The primitive CPG‐based network consisting of Hopf oscillators can generate sinusoidal signals with continuously adjustable phases in a spontaneous manner, which exactly reproduces the phase‐coordinated gait of the metameric earthworm‐like robot^[^ ^7^, ^9^ ^]^ in a decentralized way. Note that the ascending and descending phases of the sinusoidal signal are symmetric about time, corresponding to the symmetric relaxation and contraction processes of the robot segment; hence, the associated gaits are referred to as temporal‐symmetric (TS) phase‐coordinated gaits (Figure 2c, detailed in Note S2 and Figure S2, Supporting Information). To break such symmetry, the first introduced functional component is a temporal transformation (Figure 2b, detailed in Experimental Section), which is employed to tune the time‐domain features of the output signal *u_i_*. Symmetry breaking is achieved by introducing two positive scale factors, $r_{ij}^{a}$ and $r_{ij}^{d}$, into the ascending and descending phases. Specifically, $r_{ij}^{a}$ and $r_{ij}^{d}$ act on the two half‐periods of the *j* ^‐th^ cycle of *u_i_* to regulate the elapsed time in the ascending and descending phases. Hence, the degree of asymmetry of the output signals can be easily modulated by the ratio $r_{ij}=r_{ij}^{a}/r_{ij}^{d}$ in different cycles. The above temporal transformation, which essentially changes the temporal symmetry of the robot segment's deformation, would greatly expand the design space of gaits, although the fundamental mechanism of robot locomotion remains the phase coordination. Hereafter we refer to these new gaits with asymmetric ascending and descending phases as temporal‐asymmetric (TA) phase‐coordinated gait (Figure 2c).

Another type of control method for metameric robots is the discrete gaits, where a sequence of discrete numbers representing the body segment states is used to control the robot locomotion^[^ ^4^, ^45^, ^46^ ^]^ (Figure 2c, detailed in Note S3 and Figure S3, Supporting Information). The discrete gaits can also be generated via the control framework by exploiting another functional component, the activation function. Fundamentally, the activation is implemented by the Heaviside step function, which has a pivotal role in biochemistry and neuroscience.^[^ ^47^ ^]^ The activation function maps the continuous temporal‐transformed signals, symmetric or asymmetric, into step signals in the time domain. Specifically, the variation tendency of the signal, mathematically described by the time derivative of *u_i_*, i.e., d*u_i_*/d*t_i_*, is specified as the argument of the activation function. Hence, the state sequence of the segments can be prescribed by a binary vector *w_i_*, the value of which is “0” for a negative argument (i.e., descending phase) and “1” for a positive argument (i.e., ascending phase) (Figure 2b, detailed in Experimental Section), representing the fully‐relaxed state and the fully contracted state of the robot segment, respectively. Note that the distortion effect of *r_ij_* on continuous dynamics is subtly applied to discrete dynamics to regulate the ratio of the anchoring and the resting phases of a segment within a cycle (see discussion in Note S3, Supporting Information). With the combined effect of the CPG‐based network, the temporal transformation, and the activation function, all possible discrete gaits of an eight‐segment earthworm‐like robot (G1–G14, see Table S1, Supporting Information), which were generated by intuitively mimicking the retrograde peristalsis wave of the earthworm,^[^ ^4^, ^48^ ^]^ can be replicated, even including the unorthodox gaits G11 and G12.

Thus far, a versatile CPG‐inspired control framework (Figure 2) has been proposed. By sequentially performing temporal transformation and continuous‐discrete signal mapping on the output of the Hopf oscillators, the control framework not only unifies the two existing gait generators for restoring the temporal‐symmetric phase‐coordinated gaits and the discrete gaits, but also features the ability to generate novel gaits with asymmetrical waves. Figure 2c illustrates the three qualitatively different gaits originating from a single output of the CPG‐based network. In what follows, in generating the phase‐coordinated gaits, an identical phase difference $\varphi_{i}^{i+1}=-\varphi_{i+1}^{i}=\Delta \varphi (i=1,\text{…},N-1)$ is set for all bidirectional coupling between adjacent oscillators in the CPG‐based network ($\varphi_{1}^{N}=2\pi +\left(N-1\right)\Delta \varphi$), and an identical ratio *r_ij_* = *r* is set for all oscillator outputs *u_i_*. In this way, no operation is made in the spatial domain such that the actuation signals for all robot segments maintain the same waveform (i.e., the same ratio of the contraction to the relaxation process) in all cycles and are sequentially shifted by a fixed phase difference between adjacent segments. On the other hand, in generating discrete gaits, operations on both the spatial and temporal domains become a necessity. The spatial operation is achieved by taking different values of $\varphi_{i+1}^{i}$ and *r_ij_* for different *u_i_*, and additional temporal operation may be executed by taking different values of *r_ij_* for different cycles. (see details in Note S3 and Table S1, Supporting Information).

### 2.3 Effective Rectilinear Locomotion Control

**Figure 3:**
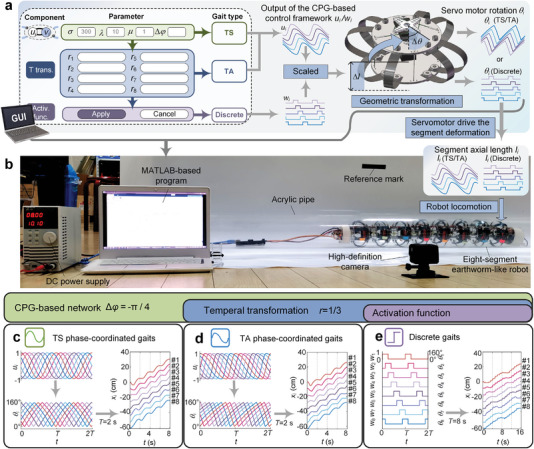
Rectilinear locomotion experiments based on the proposed control framework. a) Parameter assignment, signal generation, and geometric transformation implemented in a MATLAB‐based program. The servomotor rotation angle *θ*_*i*_ output from the program is used to actuate the robot segments by altering the axial length *l_i_*. b) The experimental setup. c–e) The control signals corresponding to the three gaits generated by the CPG‐based framework, including *u_i_* or *w_i_*, *θ*_*i*_, and the displacement‐time histories of the robot segments. The actuation period is *T* = 2s for the TS and TA phase‐coordinated gait, and *T* = 8s for the discrete gait.

We first demonstrate the effectiveness of the CPG‐based control framework in controlling the rectilinear locomotion of the earthworm‐like robot. As a test platform, an eight‐segment in‐pipe robot that mimics the morphological characteristics of the earthworm is designed and prototyped (see details in Experimental Section and Figure S4, Supporting Information). The proposed CPG‐based control framework is implemented on a MATLAB‐based program, and a user interface is designed to specify the parameters of the CPG‐based network and the functional components. Depending on the parameters, the control framework can generate qualitatively different rhythmic outputs *u_i_* or *w_i_*, which are scaled according to the axial deformation of the robot segment Δ*l_i_*. Considering that the robot segment is actuated by a servomotor, the outputs *u_i_* or *w_i_* are converted to the rotation angle *θ*_*i*_ in practice based on the geometric relation between the servomotor rotation (*θ*_*i*_) and the robot segment deformation (Δ*l_i_*) (**Figure** **3**a, detailed in Note S4 and Figure S5, Supporting Information). When the servomotors receive the signals *θ*_*i*_ and actuate accordingly, the axial length of each segment *l_i_* is changed, and the robot as a whole can make earthworm‐like locomotion inside a transparent acrylic pipe, which is recorded by a high‐definition camera for motion analysis (Figure 3b).

Based on the above experimental setup, the robot's locomotion is then tested to illustrate the effectiveness of the versatile control framework (see details in Experimental Section). Specifically, with the same parameters of the Hopf oscillators (provided in Note S1, Supporting Information) and an identical phase difference Δ*φ* = −*π*/4, three qualitatively different gaits are generated by tailoring the functional components. When none of the functional components is activated, the framework outputs a TS phase‐coordinated gait (Figure 3c); when the temporal transformation component is triggered, by setting the ratio of the ascending phase to the descending phase as *r* = 1/3, a TA phase‐coordinated gait can be acquired (Figure 3d); when both the temporal transformation (*r* = 1/3) and the activation function components are applied, the discrete gait G1 is obtained (Figure 3e). Hence, without switching the control algorithm, the robot can move through the pipeline effectively under the three qualitatively different gait, and the corresponding displacement‐time histories *x_i_* (*t*) of the robot segments are experimentally measured and demonstrated in Figure 3c–e, Figure S6 and Movie S1, Supporting Information. This thus demonstrates experimentally the effectiveness of the proposed CPG‐based control framework in unifying the three gait types and controlling the robot locomotion.

### 2.4 Locomotion Performance Improvement by Exploiting Temporal Asymmetry

Locomotion performance is critical for the earthworm‐like robot. We then expect to further improve the average steady‐state velocity of the robot by searching for the optimal gait in an expanded library. Previous research has indicated that the robot can achieve a higher maximum average velocity under the TS phase‐coordinated gaits than the achievable maximum under the discrete gaits;^[^ ^49^ ^]^ moreover, with the proposed control framework, the TS phase‐coordinated gaits are special cases of the TA phase‐coordinated gaits with *r* = 1. As a result, in what follows, the TA phase‐coordinated gaits, which are newly generated and unique for the proposed CPG‐based control framework, are examined theoretically and experimentally to show whether the temporal asymmetry could contribute to the locomotion performance, with the TS phase‐coordinated gaits as the baseline. For reference purposes, locomotion tests with the discrete gaits are presented in Note S5 and Figure S7, Supporting Information.

**Figure 4:**
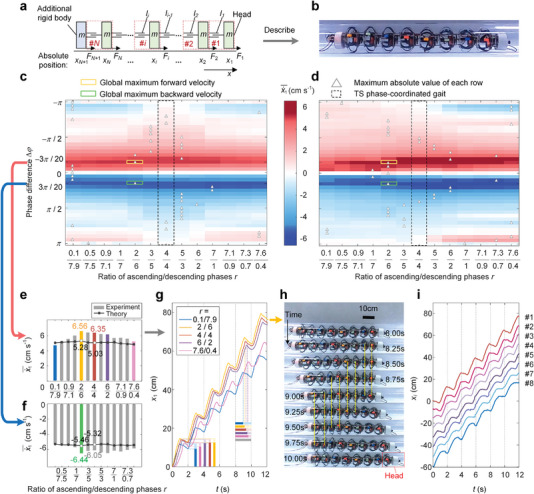
Experiment result in optimizing TA phase‐coordinated gaits. a) The dynamic model of an *N*‐segment metameric earthworm‐like robot. b) The eight‐segment earthworm‐like locomotion robot. c,d) Contour plots of c) the theoretical and d) experimental average velocity of the robot ($\bar{{\dot{x}}_{1}}$) in the Δ*φ* − *r* plane. Red and blue respectively represent forward and backward locomotion, and darker color indicates higher velocity. e,f) The average velocities of the robot corresponding to different values of *r* when Δ*φ* = −3*π*/20 and Δ*φ* = 3*π*/20; the theoretical predictions are denoted by lines, and the experimental results are denoted by bars. g) The displacement‐time histories of the robot corresponding to five TA phase‐coordinated gaits selected from e. The horizontal dark and light bars indicate the forward and backward motions of the robot in a cycle, respectively. The vertical dark and light bars indicate the net displacements and backward displacement of the robot in a cycle, respectively. h) The snapshots of the moving robot when controlled by the global optimum gait (*r* = 2/6 and Δ*φ* = −3*π*/20) corresponding to the maximum forward average velocity. i) The displacement‐time histories of the eight robot segments when controlled by the globally optimum gait.

Theoretical explorations are carried out based on the dynamic model (depicted in **Figure** **4**a and Figure S8, Supporting Information) of the eight‐segment earthworm‐like robot. The model consists of *N* segments connected via ideal displacement actuators, which are assumed to precisely control the distance between adjacent rigid bodies. Combined with the Coulomb dry friction influenced by the radial expansion of the segments, the robot system can be reduced to a single degree‐of‐freedom dynamical model subject to *n* actuation forces and *n* friction forces, governed by

(1) $$m{\ddot{x}}_{1}=\frac{1}{n+1}\left(m\sum_{i=1}^{n+1}\sum_{j=1}^{i-1}\Delta {\ddot{l}}_{j}+\sum_{i=1}^{n}F_{i}\left({\dot{x}}_{i}\right)\right)$$

where *x_i_* describes the absolute position of each rigid body, and the head displacement *x*_1_ is employed to characterize the displacement of the robot as a whole. Δ*l_j_* is the axial deformation of the robot segment, which is related to the outputs of the CPG‐based control framework, and *F_i_* is the resistance force acting on the rigid body. Detailed derivation of the governing Equation (1) can be found in Note S6, Supporting Information. Note that certain robot segments will be radially expanded during locomotion to increase the normal force between the segment and the inner wall of the pipe, thereby improving the resistance force to achieve anchor. To accurately capture the locomotion dynamics, the resistance forces applied to the robot segment at the fully contracted and the fully‐relaxed states are measured experimentally (detailed in Figure S9, Supporting Information), and the instant of contact between the segment and the inner wall of the pipe (i.e., the instant when the normal force starts to grow) is determined by recording and analyzing the radial deformation process of the segment. Based on this, a deformation‐dependent resistance force model can be determined (detailed in Note S7, Supporting Information), which is non‐smooth in nature due to the intrinsic Coulomb's dry friction at the contact. As a result, the robot becomes a non‐smooth dynamical system, whose locomotion behavior is examined through numerical and experimental means in this research.

The axial deformation Δ*l_i_* of the robot segment, which serves as the input of the dynamic model, should be sufficiently accurate to quantitatively predict the robot locomotion performance. However, generally, given the control signal *θ*_*i*_, the servomotor‐driven robot segment may not be able to accurately output the desired axial deformation $\hat{\Delta l_{i}}$ (which is obtained by geometric calculation given in Note S4, Supporting Information) due to insufficient motor capability. As a result, rather than using $\hat{\Delta l_{i}}$, the experimentally measured axial deformations associated with the control signals *u_i_*, i.e., Δ*l*_*i* − *E*_, are used as the input to the dynamic model in the numerical analysis. Specifically, for 25 different values of the ratio *r*, 25 groups of *u_i_* and the associated servomotor signals *θ*_*i*_ are generated to actuate the robot segments, and the axial deformation of each robot segment is measured via a laser displacement sensor (detailed in Note S8 and Figure S10, Supporting Information). Averaging the measurements of the eight segments yields the measured curve of the axial deformation Δ*l_E_*, from which the actual ratio *r_E_* and the magnitude of Δ*l_E_* can be calculated (listed in Table S2, Supporting Information). It reveals that both the magnitude of the axial deformation and the reachable range of the ratio *r_E_* are notably smaller than expectations (the reasons are detailed in Note S8, Supporting Information). Based on the above measurements, 13 groups of *r_E_* and Δ*l_E_* are picked out for locomotion performance evaluation (see Table S2, Supporting Information), where the selected ratios *r_E_* are largely distributed uniformly and increasing monotonically. To ensure the deformability of all segments for all values of *r_E_*, a constant magnitude $\bar{\Delta l_{E}}=13.4\mathrm{mm}$ is chosen that is attainable in all cases. By normalizing Δ*l*_E_ to the constant magnitude $\bar{\Delta l_{E}}$ and by shifting Δ*l*_E_ by the prescribing phase difference Δ*φ*, the input for each segment (Δ*l*_*i* − E_) can be obtained (Figure S10e, Supporting Information).

By dividing the 2D parameter plane Δ*φ* − *r* into 533 samples (Δ*φ* ∈ [ − *π*, *π*], with a step of *π*/20, and 13 values of *r* ∈ [0.1/7.9, 7.6/0.4]), comprehensive numerical and experimental examinations of the robot locomotion performance are carried out based on the theoretical model (Figure 4a and Figure S8, Supporting Information) and the earthworm‐like robot prototype (Figure 4b), respectively. With the given TA phase‐coordinated gaits and TS phase‐coordinated gaits, the robot's head displacements *x*_1_ are calculated or measured. By dividing the displacement of the head segment over five full cycles by 5*T*, Figure 4c,d respectively displays the distributions of the numerical and experimental average steady‐state velocity $\bar{{\dot{x}}_{1}}$ with respect to the phase difference Δ*φ* and the temporal asymmetry ratio *r*. It reveals that the average steady‐state velocity of the robot can be effectively tailored in both the magnitude and the direction by controlling the phase difference Δ*φ* and the ratio *r* of the TA phase‐coordinated gaits. Putting the theoretical and experimental results together for comparison, the following results deserve to be highlighted.

First, there is a consistency between theoretical and experimental results in terms of qualitative characteristics. This is reflected by the fact that for different values of the ratio *r* and the phase difference Δ*φ*, the distribution patterns of the average steady‐state velocity are approximately the same for both the theory and the experiment, and they are roughly reflectional‐symmetric about the *r* = 1 and Δ*φ* = 0 axes. Specifically, in terms of the ratio *r* (i.e., looking at Figure 4c,d column by column), 90.24% of the robot's maximum average velocity corresponding to different phase‐difference values occur at ratios not equal to 1 (i.e., TA phase‐coordinated gaits) in theoretical predictions; and a similar percentage, 92.68%, is observed in experiments. Moreover, both theoretically and experimentally, the ratio *r* corresponding to these new TA phase‐coordinated gaits with best performance is mainly concentrated at values of 2/6, 3/5, 5/3, 6/2, and at some of the boundary values. This, therefore, suggests that the conventional TS phase‐coordinated gaits are not globally optimal, and introducing temporal asymmetry into the gait is of benefit for improving the locomotion performance (For an example of the superiority of the locomotion performance of the TA phase‐coordinated gait (Δ*φ* = −*π*/20, with *r* taking different values) see Movie S2, Supporting Information). In terms of the phase difference Δ*φ* (i.e., looking at Figure 4c,d row by row), the robot cannot acquire net displacement when Δ*φ* = 0; by and large, the robot moves forward with negative phase differences (i.e., actuation of the posterior segments lags behind the anterior segments) and move backward with positive phase differences (i.e., actuation of the posterior segments is ahead of the anterior segments). The global plateaus of the average steady‐state velocity for both forward and backward locomotion locate in the strip areas around Δ*φ* = ±3*π*/20, which agree with the TS phase‐coordinated gaits.^[^ ^7^ ^]^ Note that when Δ*φ* → ±*π*, a reversal of the locomotion direction is spotted, especially when the ratio *r* is far away from 1 (see examples in Figure S11b,f, Supporting Information).

Quantitatively, the experimental results also agree well with the theoretical predictions. As an example, the rows with Δ*φ* = ±3*π*/20, i.e., the plateaus of the average velocity, are picked out for detailed examination (Figure 4e,f), which reveal a satisfactory agreement between the theoretical predictions and the experimental measurements. Figure 4g also displays the displacement‐time histories of the robot head for different values of *r* when Δ*φ* = −3*π*/20, from which, the locomotion performance improvement brought by the asymmetry can be clearly observed. Particularly, for forward locomotion, the theoretically‐predicted global maximum (5.28 cm s^−1^) and the experimentally‐measured global maximum (6.56 cm s^−1^) of the average steady‐state speed are achieved at the same parameters, i.e., *r* = 1/3, Δ*φ* = −3*π*/20; for backward locomotion, the theoretical maximum (5.46 cm s^−1^) and the experimental maximum (6.44 cm s^−1^) are also achieved at the same parameters, i.e., *r* = 1/3, Δ*φ* = 3*π*/20. Taking the global maximum as an example, sequences of video frames and the measured displacement‐time histories of the robot segments are illustrated in Figure 4h,i, respectively. Overall, the above quantitative comparison, on the one hand, demonstrates that the constructed dynamic model and the measurement‐based inputs can effectively and accurately predict the robot's locomotion performance (see Note S9 and Figure S11h, Supporting Information for error analysis), and on the other hand, confirms experimentally and theoretically that introducing into the temporal asymmetry can not only enrich the gait library but also improve the kinematic performance.

The underlying mechanism by which the temporal asymmetry can be beneficial for locomotion performance is related to several factors and is determined by the nonlinear robot‐environment coupled dynamical system. Note that the time proportion of forward movement in a motion cycle, (denoted by the horizontal bars in Figure 4g) and the asymmetry ratio *r* is positively correlated, while the net displacement achieved by the robot in a cycle (denoted by the vertical bars in Figure 4g) is not only associated with the forward displacement but also diminished by the undesired backward slippage. Past studies^[^ ^50^ ^]^ have shown that the robot's backward slippage is closely related to the number of radial‐expanded segments (i.e., anchored segments, exemplified in Figure 4h with vertical lines) and the radial deformation profile, and importantly, they are both jointly determined by the ratio *r* and the phase difference Δ*φ*. When the number of anchoring segments is small, or the radial deformation profile is not properly prescribed, the resistance force at the contact interface would become insufficient, which will cause the anchoring segments to slide backward and significantly weaken the locomotion performance. On the contrary, by properly setting the values of *r* and Δ*φ*, larger forward displacement and reduced backward slippage can be obtained, resulting in improved locomotion performance.

### 2.5 Smooth Gait Transitions and Smooth Braking and Starting of Locomotion

Given that the proposed CPG‐based framework considerably ameliorates the gait richness of the earthworm‐like locomotion robot, different gaits can be selected according to the current tasks. Here, gait selection is achieved by stepwise adjustment of the parameters. Note that smooth and continuous switching of gaits is essential to ensure the safety of the robot hardware and to achieve continuous locomotion. The oscillation pattern generated by the CPG‐based framework, manifested as the amplitude $\sqrt{\mu }$, frequency *f*, and phase difference Δ*φ* of the output signal, can be gradually modulated when the input of the CPG suffers tremendous changes, provided that the coupling strength *λ* and the convergence rate *σ* in the Hopf‐oscillator network are appropriately set. Based on this unique merit, we next explore the use of the CPG‐based control framework to achieve smooth and continuous gait switching of the earthworm‐like robot.

**Figure 5:**
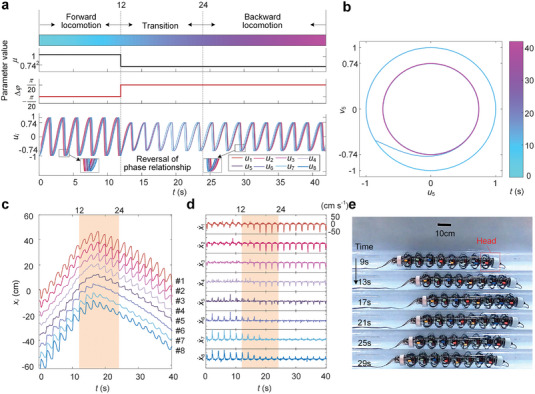
Gait transitions in the gait switching. a) Evolution of the output signals *u_i_* during the transition from a forward locomotion gait (*r* = 7/1,Δ*φ* = −*π*/20,*µ* = 1) to a backward locomotion gait (*r* = 7/1,Δ*φ* = *π*/20,*µ* = 0.74^2^). b) Evolution of the limit cycle in the *u*_5_ − *v*_5_ plane during the gait switch in a. c,d) The displacement–time histories and the velocity–time histories of the robot segments during the gait switch in a.${\dot{x}}_{i}$ is the velocity of the *i* ^th^ segment. The smooth transition stage is denoted by shades. e) The snapshots of the moving robot during the gait switching in a from *t* = 9s to *t* = 29s.

**Figure** **5**a–e illustrates an example of smooth gait switching. Specifically, by switching the parameters $\sqrt{\mu }$ (from 1 to 0.74) and Δ*φ* (from − 1*π*/20 to 1*π*/20) of the Hopf oscillators at *t* = 12 *s*, the amplitude and phase difference of the outputs *u_i_* (*i* = 1, 2, ⋅⋅⋅, 8) can be changed accordingly, thus allowing the robot to change its locomotion speed and direction. Note that although the parameters are changed in a non‐smooth stepwise way, the output signals can be smoothly evolved if the parameters *λ* and *σ* of the Hopf‐oscillator network are carefully prescribed (detailed in Note S1, Supporting Information). Figure 5a shows an obvious smooth transition process (12–24 s) between the forward locomotion phase (0–12 s) and the backward locomotion phase (24 s to the end). The essence of such a smooth signal evolution is the transient dynamics of the Hopf oscillators between two stable periodic solutions, as shown in the phase portrait of the fifth Hopf oscillator (Figure 5b), where the phase trajectory gradually evolves from the external limit cycle to the inner limit cycle, corresponding to a gradually decreasing stride of the gait. Applying the output signals to the robot, a smooth gait switch and direction reversal are observed between *t* = 12 s to *t* = 24 s in the displacement‐time histories (Figure 5c) and velocity–time histories (Figure 5d) of the robot segments, where the smooth transition stage is denoted by shades. Figure 5e also displays snapshots of the moving robot, which reveals a seamless transition from forward motion to backward motion with no upheaval or pause occurring (see Movie S3, Supporting Information for the complete switching process). Another example of gait switching from backward to forward locomotion is demonstrated in Figure S12 and Movie S4, Supporting Information.

**Figure 6:**
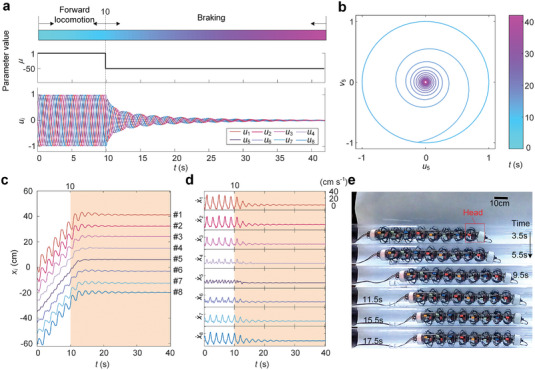
Gait transitions in the braking. a) Evolution of the output signals *u_i_* during the transition from a stable forward gait (*r* = 7/1,Δ*φ* = −*π*/4,*µ* = 1) to stop by set *µ* = −50. b) Evolution of the limit cycle in the *u*_5_ − *v*_5_ plane during the braking process in a. c,d) The displacement–time histories and the velocity–time histories of the robot segments during the braking process in a. The smooth buffering stage is denoted by shades. e) The snapshots of the moving robot during the braking process in a from *t* = 3.5 to *t* = 17.5s.

As a special case of gait switching, braking means disrupting the current steady motion and transiting to a stationary state. If there is no buffering phase before the robot stops, sharp braking will result in premature damage to robot components. To solve this problem, we can set the amplitude *µ* of the Hopf oscillators to a negative value at a certain time instant, so that the amplitude of the output signals *u_i_* (*i* = 1, 2, ⋅⋅⋅, 8) will gradually decay, and the stride of the robot will be reduced (**Figure** **6**a). Similarly, although the parameter *µ* experiences a non‐smooth stepwise switch from 1 to −50 at *t* = 10 s, careful specification of the parameters *λ* and *σ* (detailed in Note S1, Supporting Information) would allow a gradual decline in the signal amplitude, the essence of which is a smooth transition of the dynamics from a stable limit cycle to a stable fixed point (Figure 6b). Such signals would let the earthworm‐like robot gradually brake to stop from a steady motion, avoiding an abrupt stop. The smooth buffering stage is represented by the shade in Figure 6c,d, in which both the displacement and velocity amplitudes decrease evenly after *t* = 10*s*. The snapshots of the locomotion video shown in Figure 6e also validate that the robot's motion gradually slows down in a finite time until it reaches a complete stop (see Movie S5, Supporting Information for the complete braking process). Another example of gait switching, i.e., the starting process, is exemplified in Figure S13 and Movie S6, Supporting Information.

Overall, the above experiments show that our proposed CPG‐based control framework can allow the robot to seamlessly and smoothly switch gait to avoid abrupt changes, sharp stops, or starts, thus improving the adaptability of the robot in multiple scenarios. This advantage may not be significant for robots with limited working time in the laboratory, but it is crucial for extending the lifetime of robots that need to work for long periods in practical applications.

## 3 Conclusion

This study enables the successful exploration and application of neural‐inspired control on earthworm‐like robotic locomotion by proposing a generic and biologically plausible control framework. In essence, the control framework establishes and exploits the interconnections among neurobiology, collective dynamics, and bioinspired robotic locomotion. It not only unifies the existing temporal‐symmetric phase‐coordinated gaits and discrete gaits, but also generates new gaits with asymmetric waves by performing temporal transformation and continuous‐discrete signal mapping on the output of the Hopf oscillators. The capability of the framework in unifying the three gait types and spontaneously controlling the rectilinear locomotion is successfully implemented on an eight‐segment earthworm‐like robot. Through comprehensive dynamic analysis and experimental tests of the newly generated TA phase‐coordinated gaits, it is confirmed that the introduction of temporal asymmetry not only enriches the gait library but also improves the robotic locomotion performance. Furthermore, we experimentally demonstrate that the use of the CPG‐based control framework allows the robot to seamlessly switch the gait online to avoid abrupt changes, sharp stops, and starts, thus improving the robot's adaptability in various scenarios. Note that although many crawling robots with different actuations and locomotion modes have been reported in recent years;^[^ ^51^, ^52^, ^53^ ^]^ the multiple locomotion modes or gaits of the robots are relatively independent and not switchable, and their control is not incorporated into a unified framework. As a result, our research, although not necessarily as advanced as theirs in terms of robot locomotion performance, is prominently leading in terms of neural‐based control and integrated framework.

A few practical challenges still need to be addressed. The earthworm‐like rectilinear locomotion here calls for a relatively simple topology of the CPG‐based network that can be predefined by mimicking the earthworm's nervous system, but once the degrees of freedom of motion increases (e.g., turning in the plane, climbing in the space), determining the network topology is no longer as intuitive. Furthermore, for structured environments such as pipelines, open‐loop control (specifying the parameters of the CPG‐based framework) allows the earthworm‐like robot to accomplish the desired motion, but once the environment becomes complex, feedback and updating the robot's states and environmental information to the controller becomes a necessity. Implementing CPG‐based controllers in hardware and improving the efficiency of CPG‐like computation and communication is also a necessary way to deploy the CPG‐based control framework. Therefore, future research needs to be conducted from both hardware and algorithm perspectives, including exploring the multi‐objective optimization of the CPG‐based framework topology and parameters for complex motion patterns of the robot, and introducing feedback in the framework for closed‐loop neurobiologically‐inspired control of metameric robots.

## 4 Experimental Section

### Hopf Oscillators

The Hopf oscillator is governed by the following ordinary differential equations:^[^ ^37^ ^]^

(2) $$\left\{\begin{array}{c} \dot{u}=-2\pi fv+\sigma \left(\mu -u^{2}-v^{2}\right)u\\ \dot{v}=2\pi fu+\sigma \left(\mu -u^{2}-v^{2}\right)v \end{array}\right.$$

*f* and $\sqrt{\mu }$ specify the oscillation frequency and the amplitude, respectively, and *σ* is a constant controlling the convergence speed of *u* and *v* to the limit cycle, as shown in the phase portrait in Figure S1a,b, Supporting Information.

The dynamics of the proposed CPG network are described by the following model:^[^ ^35^ ^]^

(3) $$\begin{array}{c} \left[\begin{array}{c} {\dot{u}}_{i}\\ {\dot{v}}_{i} \end{array}\right]=\left[\begin{array}{cc} \sigma (\mu -u_{i}^{2}-v_{i}^{2}) & -2\pi f\\ 2\pi f & \sigma (\mu -u_{i}^{2}-v_{i}^{2}) \end{array}\right]\left[\begin{array}{c} u_{i}\\ v_{i} \end{array}\right]\\ +\lambda \sum_{j}\left[\begin{array}{cc} \cos\varphi_{j}^{i} & -\sin\varphi_{j}^{i}\\ \sin\varphi_{j}^{i} & \cos\varphi_{j}^{i} \end{array}\right]\left[\begin{array}{c} u_{j}\\ v_{j} \end{array}\right],\left(\begin{array}{c} i=1,j=2\\ i=2,3,\text{…},7,j=i\pm 1\\ i=8,j=1,7 \end{array}\right) \end{array}$$

where *λ* represents the coupling strength between oscillators. $\varphi_{j}^{i}=-\varphi_{i}^{j}=\varphi_{i}-\varphi_{j}$ denotes the phase difference of the *i‐*th oscillator relative to the *j*‐th oscillator. Without loss of generality, $\varphi_{j}^{i}\in \left[-\pi ,\pi \right]$ in this work. Figure S1c, Supporting Information shows the spatiotemporal dynamics of the CPG network starting from an initial unsynchronized condition, where the oscillations synchronize rapidly in accordance with the *σ*‐determined speed and *λ*‐determined strength, and remain phase‐locked after entering the steady state, with the phase difference determined by $\varphi_{j}^{i}$.

Note that the outputs of the eight oscillators are used to control the deformations of the robot segments. Hence, to be consistent with the metameric characteristics of the earthworm, all oscillators are assumed to be identical (i.e., the initial values of *u* and *v*, the values of *f*, $\sqrt{\mu }$, and *σ* are the same) when generating a certain gait. However, when generating different gaits or performing gait switches, these parameters need to be altered. Their specific values are discussed in Note S1, Supporting Information.

### Temporal Transformations and Activation Functions

To break the temporal symmetry of the output signal, a functional component, the temporal transformation, is introduced into the control framework. Specifically, the scale factor of the ascending and descending phases, $r_{ij}^{a}$ and $r_{ij}^{d}$, act on the two half‐periods of the *j*‐th cycle of *u_i_*, i.e.,

(4) $$t_{ij}^{a}=r_{ij}^{a}T/2,t_{ij}^{d}=r_{ij}^{d}T/2\left(i=1,\text{…},8;j=1,\text{…},n\right)$$

where $t_{ij}^{a}$ and $t_{ij}^{d}$are the scaled ascending phase and descending phase of the *j‐*th cycle of oscillator *i*, and *T* = 1/*f* represents the period of *u_i_*. Hence, the asymmetry degree can be described by the ratio of the ascending phase to the descending phase, i.e.,

(5) $$r_{ij}=r_{ij}^{a}/r_{ij}^{d}\left(i=1,\text{…},8,j=1,\text{…},n\right)$$

In this way, the waveform of *u_i_* can be varied from cycle to cycle and segment to segment.

Furthermore, another functional component, the activation function, is introduced into the control framework. The activation function, which is fundamentally a Heaviside step function,^[^ ^47^ ^]^ could map a continuous signal *u_i_* to a binary vector *w_i_*, thus generating a discrete gait

(6) $$w_{i}=\left\{\begin{array}{c} 1,du_{i}/dt_{i}>0,\\ 0,\;\;\mathrm{else}, \end{array}\right.\left(i=1,\text{…},8\right)$$

In generating TS and TA phase coordinated gaits, the scale factors for all oscillator outputs are assumed to be identical, i.e., $r_{ij}^{a}=r^{a}$, $r_{ij}^{d}=r^{d}$, and *r_ij_* = *r*, such that all robot segments share the same waveform. In other words, no operation is made in the spatial domain. In addition, a constraint *r^a^* + *r^d^* = 2 is added to ensure that the period of the signal remains unchanged such that

(7) $$t^{a}+t^{d}=\left(r^{a}+r^{d}\right)T/2=T$$

However, in generating unorthodox discrete gaits, additional operations in both the temporal and spatial domains are required, which, therefore, calls for altering the scale factors $r_{ij}^{a}$ and $r_{ij}^{d}$ for different outputs *u_i_* and different cycles, see examples in Table S1, Supporting Information.

### Design and Fabrication of the Robot

Figure S4 in the Supporting Information shows the prototype of the in‐pipe earthworm‐like robot. The robot consists of eight identical segments that are connected in series via screw rods. Each segment is made up of two acrylic plates, eight spring‐steel belts, a servomotor, and two servomotor‐driven cords. The active servomotor‐driven cord and passive spring‐steel belts act similarly to the longitudinal and circular muscles of the earthworm. For detailed design and fabrication of the robot please refer to.^[^ ^4^, ^48^ ^]^ The antagonistic deformations of the two types of “muscles” in our robot are physically locked. When the servomotor is actuated forward, the cords are tensioned to axially shorten the segment and radially expand the robot due to the outward bending of the spring‐steel belts. Hence, the normal force and the friction force between the segment and the inside wall of the tube will be increased, thus achieving the anchor effect. When the servomotor is actuated in reverse, the cords are loosened such that the robot segment elongates axially and contracts radially. By executing the above actuations sequentially in a discrete gait or phase‐coordinated gait pattern, the robot as a whole can perform earthworm‐like locomotion.

The weight of a single segment is 75.3 g. A fully‐relaxed segment (the servomotor is not actuated) has the maximum axial length of 56.1 mm and the minimum radial diameter of 105.0 mm. A fully contracted segment (the servomotor rotates 160°) has the minimum axial length of 27.1 mm and the maximum radial diameter of 125.0 mm. The eight‐segment earthworm‐like robot prototype is 725.0 mm in length and 706.0 g in weight. The inner diameter of the acrylic pipe is 115.0 mm.

### Locomotion Tests of the Earthworm‐Like Robot

The control signals are generated by the CPG‐based control framework, where the gait type and the parameters are specified through the GUI. Then, according to the geometric relations between the axial deformation of the robot segment and the rotation angle of the servomotor, the output signals (*u_i_* or *w_i_*) are converted into the rotation‐angle signals for the servomotors (detailed derivation of the geometric relations can be found in Note S4, Supporting Information). The above procedures are all implemented by a MATLAB‐based program (Figure 3a). The servomotors receive the rotation commands from MATLAB via the STM32 board and actuate the robot to perform the specified gait.

The robot is asked to work within a transparent acrylic pipe that is 2 m long. The locomotion is recorded by a high‐definition camera. The displacement‐time histories of the robot segments are obtained by tracking the fluorescent markers attached at the middle of each segment, and the velocity–time histories can be obtained via the central difference method.

## Conflict of Interest

The authors declare no conflict of interest.

## Supporting information

Supporting Information

Supplemental Movie 1

Supplemental Movie 2

Supplemental Movie 3

Supplemental Movie 4

Supplemental Movie 5

Supplemental Movie 6

## Data Availability

The data that support the findings of this study are available in the supplementary material of this article.
