# Supplementary material for: A CPG‐Based Versatile Control Framework for Metameric Earthworm‐Like Robotic Locomotion
Source: Adv Sci (Weinh). 2023 Feb 12;10(14):2206336. doi: 10.1002/advs.202206336 (PMC10190653; doi:10.1002/advs.202206336)
Supplement: Supplementary file 1 — Supporting Information [file ADVS-10-2206336-s006.pdf]

ADVANCED SCIENCE
Open Access

Supporting Information

for *Adv. Sci.*, DOI 10.1002/advs.202206336

A CPG-Based Versatile Control Framework for Metameric Earthworm-Like Robotic Locomotion

*Qinyan Zhou, Jian Xu and Hongbin Fang**

Supporting Information

A CPG-based versatile control framework for metameric earthworm-like robotic locomotion

*Qinyan Zhou, Jian Xu, and Hongbin Fang**

Institute of AI and Robotics, State Key Laboratory of Medical Neurobiology, MOE Engineering Research Center of AI & Robotics, Fudan University, Shanghai 200433, China

*To whom correspondence should be addressed. Email: fanghongbin@fudan.edu.cn (H. Fang)

This file includes:

- Notes S1 to S9
- Figures S1 to S13
- Tables S1 to S2
- Captions of Movies S1 to S6
- References for SI reference citations

Other supplementary materials for this manuscript include the following:

- Movies S1 to S6

Supporting Notes

Note S1: Parameter values of the Hopf oscillator

The model of N coupled Hopf oscillators in this paper is given by:

$$\begin{bmatrix}\dot{u}_i\\ \dot{v}_i\end{bmatrix}=\begin{bmatrix}\sigma(\mu-u_i^2-v_i^2) & -2\pi f\\ 2\pi f & \sigma(\mu-u_i^2-v_i^2)\end{bmatrix}\begin{bmatrix}u_i\\ v_i\end{bmatrix}$$
$$+\lambda\sum_j\begin{bmatrix}\cos\varphi_j^i & -\sin\varphi_j^i\\ \sin\varphi_j^i & \cos\varphi_j^i\end{bmatrix}\begin{bmatrix}u_j\\ v_j\end{bmatrix},\begin{pmatrix}i=1,\ j=2\\ i=2,3,\cdots,N-1,\ j=i\pm1\\ i=N,\ j=1,N-1\end{pmatrix}, \tag{S1}$$

The parameters of the coupled Hopf oscillators are discussed as follows:

(1) μ: In terms of the dynamics of the Hopf oscillator, μ determines the whether the dynamical system can have a limit cycle. When $\mu>0$, the Hopf oscillator has a stable limit cycle which is a circle of radius $\sqrt{\mu}$ and an unstable fixed point at the origin. When $\mu<0$, the Hopf oscillator only has a stable fixed point at the origin and there is no stable limit cycle, so the state variables will spirally converge to the origin from any initial value.

In terms of the output signal of the Hopf oscillator, $\sqrt{\mu}$ denotes the amplitude of u_i and v_i, which defaults to 1. Hence, the output u_i of the oscillator can be scaled to the axial deformation of the segment (i.e., l_i) according to the maximum and minimum axial length of the segment. Besides, μ was altered from 1 to 0.74^2 to change the robot's stride in gait switching, and μ experices a switch from 1 to −50 in braking to attract u_i and v_i gradually converge to zero.

(2) u_i and v_i: In terms of the dynamics of the Hopf oscillator, u_i and v_i are state variables. when $\mu>0$, u_i and v_i with any non-zero initial value can always return to the limit cycle at $\sqrt{u_i^2+v_i^2}=\sqrt{\mu}$ (Figure S1a), while u_i and v_i with zero initial value will stay in the unstable fixed point (i.e., the origin).

In terms of the output signals of coupled Hopf oscillators, u_i and v_i are the outputs of the ith oscillator, which are sinusoidal waves with the same shape and a phase difference of $\pi/2$ (Figure S1b). As long as one of the state variables of all coupled oscillators has a non-zero initial value, these oscillators will generate periodic oscillation signals. The initial values of u_i and v_i of all oscillators are taken as –0.2 here as an example.

(3) f: In terms of the dynamics of the Hopf oscillator, $2\pi f$ denotes the angular velocity of the oscillator on the trajectory of the limit cycle.

In terms of the output signal of the Hopf oscillator, f specifies the frequency of u_i and v_i, which takes $1/2$ in generating TS phase-coordinated gaits and TA phase-coordinated gaits, and $1/8$ or $1/4$ in generating discrete gaits G1-G14. This depends on how long the robot segment is asked to return to its current state in each gait.

(4) φ_j^i and $\Delta\varphi$: $\varphi_j^i = \varphi_i - \varphi_j$ determines the phase difference of the ith oscillator to the jth oscillator, which is adjustable between [$-\pi$, π]. Here, bidirectional coupling between adjacent oscillators (with phase difference $\varphi_i^{i+1} = -\varphi_{i+1}^i$ $(i = 1, \cdots, N-1)$) and unidirectional coupling from the tail oscillator to the head oscillator (with phase difference φ_1^N) are considered. Note that $\sum_{i=1}^{N-1} \varphi_{i+1}^i + \varphi_1^N = 2\pi$ needs to be satisfied to ensure that the given phase differences are achievable. In generating the TS and TA phase-coordinated gaits, an identical phase difference $\varphi_i^{i+1} = -\varphi_{i+1}^i = \Delta\varphi$,$(i = 1, \cdots, N-1)$ is set for all bidirectional coupling between adjacent oscillators, such that the phase difference from the tail oscillator to the head oscillator is $\varphi_1^N = 2\pi + (N-1)\Delta\varphi$). In generating discrete gaits, φ_{i+1}^i corresponding to bidirectional coupling can be different (listed in Table S1, Supporting Information), but the rule $\sum_{i=1}^{N-1} \varphi_{i+1}^i + \varphi_1^N = 2\pi$ still needs to be satisfied.

(5) σ and λ: σ determines how fast the oscillation converges to the stable limit cycle, also determines the time elapsed for transients of u_i and v_i. λ represents the coupling strength between oscillators. Note that σ and λ jointly affect the output u_i, their values need to be handled carefully.

When generating a steady-state gait, the oscillations need to converge accurately and quickly to the desired limit cycle solution. If the value of σ is too small, the difference between the amplitude of the outputs u_i and the desired amplitude $\sqrt{\mu} = 1$ is significant (see Figure S1d, Supporting Information where $\sigma = 0.3$, $\lambda = 0.1$). By appropriately increasing the value of σ, the amplitude of u_i can agree with $\sqrt{\mu}$ (see Figure S1e, Supporting Information, where $\sigma = 30$, $\lambda = 0.1$). However, since the value of λ is small, the coupling between oscillators is weak, such that the phase difference between u_i does not reach the desired phase difference $\varphi_i^{i+1} = \Delta\varphi = -\pi/4$ (see Figure S1e, Supporting Information where $\sigma = 30$, $\lambda = 0.1$). By increasing the value of λ, although the phase difference between u_i can agree with $\Delta\varphi$, the amplitude of u_i deviates from the expected amplitude $\sqrt{\mu}$ again(see Figure S1f, Supporting Information where $\sigma = 30$, $\lambda = 10$). The above attempts indicate that the value of σ and λ need to be considered holistically. Based on our experience, σ =300 and λ =10 are taken to ensure fast convergence and stable coupling of the oscillations (Figure S1g, Supporting Information).

On the other hand, for achieving gait transitions, instead of fast and accurate convergence, we need a progressive and smooth convergence. After several attempts we found that smaller σ and λ can elongate the transient phase that is required in these cases. Specifically, the smaller σ allows the state variables of individual oscillators to have sufficiently long spiral paths between limit cycles or between a limit cycle and the origin, which enables a smooth switching of the amplitude of the output signal. Similarly, the smaller λ ensures that the

oscillators have a long enough and smooth coupling process before reaching a new stable coupling relationship. Hence, we take $\sigma = 4.5$, $\lambda = 0.3$ in gait switching (see Figure 5 and Figure S12, Supporting Information), $\sigma = 0.021$, $\lambda = 0.5$ in braking (see Figure 6), and $\sigma = 0.002$, $\lambda = 0.2$ in starting (see Figure S13, Supporting Information).

Note S2: TS phase-coordinated gait control

Fang et.al has proposed an approach to control the locomotion of metameric robots via phase coordination[1] (see Figure S2a, Supporting Information). Specifically, the constituent segments of a metameric earthworm-like robot are connected via ideal displacement actuators, such that the distance between adjacent segments is controlled by $l_i\left(t\right)=x_i-x_{i+1}=L_0+\Delta l\left(t,\varphi_i\right)$, $\left(i=1,\ldots,n\right)$, where x_i describes the absolute position of each segment, L_0 denotes the original length between adjacent segments. $\Delta l\left(t,\varphi_i\right)$ is the axial oscillatory deformation of the actuator, which is assumed to change harmonically, expressed as $\alpha L_0 \sin\left(\omega t+\varphi_i\right)$, where αL_0 is the oscillation amplitude with $0<\alpha<1$. When continuous viscous resistance force is applied, analytical solution of the average locomotion speed of the robot can be derived under sinusoidal wave-based phase-coordinated gait, so that the global optimum can be determined through optimization method. When Coulomb's dry friction is applied, the global optimum is again determined through traversing the parameters based on prespecified grids. It has been demonstrated that the actuation phase differences are directly related to the average velocity. By optimizing the phase-difference pattern based on their symmetric properties, it has been discovered that identical-phase-difference (IPD) pattern (see Figure S2b, Supporting Information) is preferred for implementation because it not only achieves an average steady-state velocity that is very close to the global optimum and greatly reduces the number of independent variables.[1] Specifically, the IPD pattern is given by

$$\varphi_1=0,\varphi_i=\varphi_1+\left(i-1\right)\Delta\varphi,(i=1,\cdots,n). \tag{S2}$$

Note S3: Discrete gait control

In discrete gait control[2], each robot segment works similarly to the earthworm's body segment. Each robot segment possesses two states, the fully-relaxed and the fully-contracted states, denoted by '0' and '1', respectively. In each transition, the robot segment can exhibit four types of switching action, namely, contracting (0→1), relaxing (1→0), temporarily anchoring (1→1), and resting (0→0) (see Figure S3a, Supporting Information). The time required by a switching action is Δt, and Δl is the deformation of a robot segment achieved when switching between '1' and '0' states.

Without loss of generality, we assume that there are k driving modules in an N-segment earthworm-like robot. A driving module consists of n_C contracting segments, n_A anchoring segments, and n_R relaxing segments (see Figure S3a, Supporting Information). During a transition, the anchoring segments temporarily anchor with the environment and remain stationary, the contracting segments pull themselves and the posterior segments forward, and the relaxing segments push themselves and the anterior segments forward. In addition, $n_\mathrm{C} = n_\mathrm{R}$ is required to obtain a steady-state locomotion. With different values of k, n_A and n_R, the robot will display different gaits. Therefore, the parameters $(N, k, n_\mathrm{A}, n_\mathrm{R})$ completely determine the state of all segments and the gait of the N-segment robot. In previous research[2], a kinematic model of an N-segment earthworm-like robot has been developed, and the average velocity is derived as

$$\bar{V} = \frac{N - k\left(n_\mathrm{A} + n_\mathrm{R}\right)}{N / n_\mathrm{R}} \frac{\Delta l}{\Delta t}. \tag{S3}$$

In this work, the theoretical velocity of the robot corresponding to a certain discrete gait of the robot is calculated via Equation S3.

Based on the retrograde peristalsis wave principle, 14 gaits (denoted as G1 to G14) can be generated when N=8 [3], listed in Table 1 in the Supporting Information, and G9 is exemplified in Figure S3b in the Supporting Information. With the proposed control framework in this paper, in order to generate the binary vector w_i corresponding to the 14 discrete gaits, the values of φ_j^i, r_{ij}^a, and r_{ij}^d are given in Table S1 in the Supporting Information. Similar to the TA phase-coordinated gait, the degree of asymmetry of the '0' phase and '1' phase is modulated by the ratio $r_{ij} = r_{ij}^\mathrm{a} / r_{ij}^\mathrm{d}$, schematically illustrated in the last column of Table S1, Supporting Information.

Particularly, for discrete gaits G7 and G9, r_{ij} need to take different values for odd oscillators (i=1, 3, 5, 7) and even oscillators (i=2, 4, 6, 8), which is fundamentally an operation in the spatial domain. For discrete gaits G11 to G14, the ratio r_{ij} takes different values for different cycles of u_i, which is fundamentally an additional operation in the temporal domain.

Note S4: Geometric relations between the axial deformation Δl_i of the robot segment and the rotation angle $\Delta\theta_i$ of the servomotor

Figure S5 of the Supporting Information shows the geometric parameters a_0, b, D, d, h for deriving the geometric relations. For the prototyped robot segments in this research, the values of these parameters are: $a_0 = 18.55$ mm, $b = 2.33$ mm, $D = 32.13$ mm, $d = 15.31$ mm, $h = 12.05$ mm. These parameters are identical for all segments and are used to derive the geometric relation between the axial deformation Δl_i of the robot segment and the rotation angle $\Delta\theta_i$ of the servomotor Specifically, l_i is the axial length of the ith segment. When the servomotor is not actuated, the segment is of its maximal length $l_{\max}$. When the servomotor rotates by $\Delta\theta_i$, the length of the cord exposed above the acrylic changes from a_0 to a_i, which then induces a change in the axial length of the robot segment Δl_i. Δl_i and a_i can be expressed by

$$\Delta l_i = l_{\max} - l_i = a_i - a_0 \tag{S4}$$

The projected length of a_i on the horizontal plane (i.e., c_i) can be calculated via

$$c_i = \sqrt{a_i^2 - h^2}. \tag{S5}$$

For the simplicity of the calculations, we assume, within the margin of error, that

$$s_i = c_i + b. \tag{S6}$$

Using the law of cosine, we have

$$\Delta\theta_i = \arccos\left(\frac{D^2 + d^2 - s_i^2}{2Dd}\right). \tag{S7}$$

Substituting Equation S4, Equation S5 and Equation S6 into Equation S7, $\Delta\theta_i$ yields

$$\Delta\theta_i = \arccos\left[\frac{D^2 + d^2 - \left(\sqrt{\left(l_{\max} - l_i + a_0\right)^2 - h^2} + b\right)^2}{2Dd}\right]. \tag{S8}$$

At the initial fully-relaxed state, the servomotor is not actuated, hence, the rotation angle of the servomotor is

$$\theta_i = \Delta\theta_i \tag{S9}$$

Note S5: Locomotion performance under discrete gaits

Figure S7 in the Supporting Information shows the theoretical and experimental results of the average velocity of the robot under the 14 discrete gaits. Theoretical predictions are obtained based on Equation S3, and the experimental values are calculated by dividing the displacement into 3 periods by the time. Some conclusions are worth mentioning.

(1) In general, the theoretical average velocity of the robot shares the same qualitative trend as the experimental average velocity. Both the theoretical and experimental maximums are achieved at gait G11. Quantitatively, however, the theoretical average velocity is always higher than the experimental one. This is because the ideal anchor and ideal actuation assumptions in the kinematic model cannot be satisfied in experiments.

(2) Increasing the number of the driving module (i.e., k) will reduce the average locomotion velocity of the robot. This is because the coexistence of multiple driving modules will limit the propagation of displacement through the robot body.

(3) With the same number of driving modules (i.e., k) and the same number of anchoring segments (i.e., n_A), more contracting/relaxing segments (i.e., a larger value of n_R) in the driving module enable higher average velocity of the robot. However, meanwhile, the differences between the experimental and theoretical results are also greater. The large deviations come from the undesired backward slippage of the anchoring segments, which becomes severer when there are more contracting/relaxing segments. The underlying nature of such slippage is the stick-slip motions, which have been discussed in detail in[4]. Particularly, the largest deviations occur in gaits G11 and G12.

(4) With the same number of driving modules (i.e., k) and the same number of contracting/relaxing segments (i.e., n_R), more anchoring segments (i.e., a larger value of n_A) in the driving module will bring down the average velocity of the robot. However, the differences between the experimental and theoretical results become smaller because more anchoring segments can suppress the backward slippage.

Note S6: Detailed derivation of the dynamic model of the earthworm-like robot

Figure 4a and Figure S8 displays the equivalent dynamic model of the robot prototyped in this research. The model consists of *n* segments connected via ideal displacement actuators, which are assumed to be able to accurately control the distance between adjacent bodies. In detail, the axial deformation of the robot segment Δl_i is first related to the outputs (u_i or w_i) of the CPG-based control framework via the following equation

$$L_0 = (l_{\max} + l_{\min})/2,$$
$$\Delta l_i = \begin{cases} L_0 + u_i(l_{\max} - l_{\min})/2, & \text{TS / TA phase-coordinated gait} \\ L_0 + (2w_i - 1)(l_{\max} - l_{\min})/2, & \text{discrete gait,} \end{cases} \tag{S10}$$

where $l_{\min}$ and $l_{\max}$ denote the axial length of the segment in the fully-contracted state and the fully-relaxed state, respectively, and L_0, the midpoint of $l_{\min}$ and $l_{\max}$, is introduced for coordinate translation. $u_i \in [-1,1]$ and $w_i \in \{0,1\}$ are the control signals output from the CPG-based control framework for the TS/TA phase-coordinated gaits and the discrete gaits, respectively. Hence, the distance between adjacent bodies l_i can be expressed by $l_i = L_0 + \Delta l_i$, and the displacement and velocity of each rigid body yield

$$x_i = x_1 - \sum_{j=1}^{i-1} l_j,$$
$$\dot{x}_i = \dot{x}_1 - \sum_{j=1}^{i-1} \dot{l}_j \, (i = 1, 2, \cdots, N+1), \tag{S11}$$

where x_i describes the absolute position of each body, with x_1 and x_{N+1} representing the displacement of the head and the tail, and the corresponding velocity is denoted by the first-order derivative of x_i.

In this research, the head displacement x_1 and the head velocity $\dot{x}_1$ are respectively employed to characterize the displacement and velocity of the robot as a whole. Hence, the dynamic equation of each rigid body gives

$$m\ddot{x}_1 = \mu_{i-1} - \mu_i + F_i \;\; (i = 1, \cdots, N+1), \tag{S12}$$

where μ_i and μ_{i-1} are the inner forces generated by the *i*-th and (*i*-1)-th actuators, with $\mu_0 = \mu_{n+1} = 0$, F_i is the resistance force acting on the body, which will be modeled in Note S7, Supporting information. Substituting Equation S10 and Equation S11 into Equation S12 and summing up the dynamic equation for all rigid bodies yields the governing equation of the robot's locomotion

$$m\ddot{x}_1 = \frac{1}{n+1}\left(m\sum_{i=1}^{n+1}\sum_{j=1}^{i-1} \Delta\ddot{l}_j + \sum_{i=1}^{n} F_i(\dot{x}_i) \right). \tag{S13}$$

Note S7: Modelling of the resistance force

When the earthworm-like robot works inside a pipe, the resistance force between the robot segment and the pipeline inwall is closely related to the segment's radial diameter. In addition, similar to the earthworm's body segment, the radial and axial deformations of the robot segment are interrelated. Taking this into consideration, the resistance force is modeled as

$$\begin{aligned} &F\left(l_i, \dot{x}_i\right) = q\left(l_i\right) f\left(\dot{x}_i\right), \\ &q\left(l_i\right) = 1 + \alpha\left(1 - \tanh\left(\beta\left(l_i - l_{\min}\right)\right)\right), \end{aligned} \tag{S14}$$

where $F\left(\dot{x}_i\right)$ is the resistance force acting on the i-th rigid body, $f\left(\dot{x}_i\right)$ denotes the friction force model at the contact surface. In this research, isotropic friction contact is assumed, and Coulomb's dry friction $f\left(\dot{x}_i\right) = \xi mg \cdot \mathrm{sgn}(\dot{x}_i)$ is considered, where ξ is the Coulomb dry friction coefficient. The parameter $q\left(l_i\right)$ essentially characterizes the effect of the radial dimension (interrelated with the axial dimension l_i) of the robot segment on the resistance force by employing the tanh function. Here, $\left(l_i - l_{\min}\right)$ not only describes the change of the axial length of the i-th segment, but also equivalently describes the change of the radial dimension. When the robot segment stays in the fully-relaxed state, i.e., $l_i = l_{\max}$, the robot segment is not in full contact with the inner wall of the pipe, and the resistance force should be identical to the Coulomb's dry friction force. As the robot segment gradually expands in the radial direction, the segment's axial length gradually decreases. Until the robot segment is in full contact with the inner wall of the pipe, the resistance force is always very close to the Coulomb's dry friction. Define the length of the segment when it is in full contact (or loses fully contact) with the inner wall of the pipe as l_{contact} ($l_{\min} < l_{\text{contact}} < l_{\max}$). At this critical instant, we ask $q\left(l_{\text{contact}}\right) = 1 + \alpha\left(1 - \tanh\left(\beta\left(l_{\text{contact}} - l_{\min}\right)\right)\right) \to 1$, which can be achieved by appropriately prescribing the value of β. Keep contracting the segment, the segment cannot be further expanded in the radial direction due to the constraint of the inner wall; rather, the segment imposes a large extrusion force to the wall, which thus greatly increases the resistance force. When the segment reaches its minimum length, i.e., $l_{\min}$, q takes the maximum value $1+\alpha$.

To determine the values of the coefficients α, β, and ξ in the resistance force model, the resistance forces are measured when the segment is in the fully-relaxed state (F_{R}) and the fully-contracted state (F_{C}). Figure S9a, Supporting Information displays the experimental setup. Considering that the diameter of the fully-relaxed and fully-contracted segments is 105.0 mm and 125.0 mm, respectively, we chose an acrylic pipe with an outer diameter of 125.0 mm and an inner diameter of 115.0 mm. The segment was pulled through the acrylic pipe with a tensiometer at a constant speed of 5 mm/s for more than 15 seconds, and the data is recorded with a sampling frequency of 10 Hz. By fitting the force curve recorded by the tensiometer with

a horizontal line, the resistance force can be obtained. Taking the average of five experiments for the fully-relaxed and the fully-contracted segments, respectively, we have $F_{\mathrm{R}} = 0.3361$ N and $F_{\mathrm{C}} = 3.4398$ N (see Figure S9b, Supporting Information). The mass of a single segment is *m*=75.3g (see Methods). Substituting F_C, F_R, and m into Equation S15

$$\begin{aligned} F_{\mathrm{C}} &= (1+\alpha) f\left(\dot{x}_i\right) = (1+\alpha)\xi mg \cdot \mathrm{sgn}(\dot{x}_i), \\ F_{\mathrm{R}} &= f\left(\dot{x}_i\right) = \xi mg \cdot \mathrm{sgn}(\dot{x}_i), \end{aligned} \tag{S15}$$

from which, $\xi = 0.4555$ and $\alpha = 9.2345$ can be obtained. Moreover, by recording the radial expansion process with a high-definition camera, we have $l_{\mathrm{contact}} = 115.0$ mm. We set $\beta = 200$ to meet the requirement. It shows that $q\left(l_{\mathrm{contact}}\right) = 1.0737$ and $q\left(l_{\max}\right) = 1.0003$, which satisfy our requirements that $q\left(l_{\mathrm{contact}}\right)$ and $q\left(l_{\max}\right)$ approach to 1.

Figure S9c, Supporting Information displays the variation of q with respect to t by assuming that the axial length l_i varies with time following a sinusoidal low, from which the stages with and without full contact between the robot segment and the inner wall of the pipe are indicated. Figure S9d, Supporting Information shows the relationship between q and l_i, and the relationship between $F\left(\dot{x}_1\right)$ and l_i in the obtained resistance force model.

Note S8: Axial deformations of the robot segments

To quantitatively predict the locomotion performance of the robot based on the dynamic model, the input, i.e., Δl_i, need to be determined accurately. To this end, 25 values of r are taken in the form of $i/(8-i)$, and some extreme values of r are taken to demonstrate the temporal asymmetry. Specifically, the 25 values of r are taken by letting $i = 0.1, 0.2, \cdots, 0.9; 1, 2, \cdots, 7; 7.1, 7.2, \cdots, 7.9$. Corresponding to them, 25 groups of u_i and the associated servomotor signal θ_i are generated to actuate the robot segment segments.

Figure S10, Supporting Information displays the experiment setup for measuring the axial deformation of each robot segment. In detail, for each value of r, the servomotor rotation θ_i is derived from the output u_i by the geometric transformation. We fix one robot segment on the frame at a time and actuate it via the servomotor rotation θ_i, the axial deformation of this segment is measured by the laser displacement sensor. The above operations are performed sequentially for eight robot segments (Figure S10a and Figure S10b, Supporting Information). Each segment is actuated continuously for more than five cycles (the period is set to 2s). By averaging the measurements of the eight segments, the measured axial deformation curve Δl_E can be obtained (Figure S10c and Figure S10d, Supporting Information). The measured value of the ratio, i.e., r_E, can be obtained by calculating the ratio of ascending phases to the descending phases (Figure S10d, Supporting Information).

Table S2 in the Supporting Information lists the measured magnitude of the axial deformation Δl_{E} and the measured value of the ratio r_{E}. It shows that the reachable range of r_{E} and Δl_{E} are notably smaller than the expected values. If $r < 1$, the measured value $r_{\mathrm{E}} > r$ (e.g., in Figure S10a and Figure S10d in the Supporting Information $r = 1/7$, and the measured value is $r_{\mathrm{E}} = 1/3.6203$); on the contrary, if $r > 1$, the measured value $r_{\mathrm{E}} < r$. The measured magnitude of Δl_{E} is also smaller than expected (e.g., in Figure S10a and Figure S10d in the Supporting Information, with $r = 1/7$, the magnitude for Δl_i is 14.5 mm, while the measured magnitude for Δl_{E} is only 14.2 mm). The above deviations come from insufficient motor capability. Specifically, the reachable range of r_{E} is significantly smaller than the expected value because the spring-back speed of the steel belts is not sufficient to bring the robot from a fully-contracted state to a fully-relaxed state in a very short time (corresponding to a very small value of r), and the rotation speed of the servo motor is not sufficient to actuate the robot from a fully-relaxed state to a fully-contracted state in a very short time (corresponding to a very large value of r). The measured amplitude Δl_E is also smaller than expected because at a particular value of r, the servo motor reverses before it reaches the specified maximum rotation angle, and thus the expected amplitude of robot deformation is not reached.

13 groups of r_{E} and $\Delta l_{i-\mathrm{E}}$ are picked out for locomotion performance evaluation (see Table S2, Supporting Information). To ensure the deformability of all robot segments for all values of r_{E}, a constant magnitude $\overline{\Delta l_{\mathrm{E}}} = 13.4\ \mathrm{mm}$ is chosen that is attainable in all cases. By normalizing Δl_{E} to the constant magnitude $\overline{\Delta l_{\mathrm{E}}}$ and by shifting $\overline{\Delta l_{\mathrm{E}}}$ by the prescribing phase difference $\Delta\varphi$, the input for each segment ($\Delta l_{i-\mathrm{E}}$) can be obtained (Figure S10e, Supporting Information). Corresponding to the constant magnitude $\overline{\Delta l_{\mathrm{E}}} = 13.4\ \mathrm{mm}$ and based on the geometric relation given by Equation S8, the maximum rotation angle of the servomotor is set to 160°.

Note S9: Discussions on the deviations between the experimental and theoretical average steady-state velocity of the robot

By observing the theoretical and experimental locomotion performances in Figure 4c and Figure 4d, as well as the errors between them in Figure S11g in the Supporting Information, there are also some deviations between the experiment and the theory that need attention. For example, if we focus on the maximum average steady-state velocity corresponding to each phase-difference value (denoted by the triangle in each row), the experimental results are not necessarily identical to the theoretical prediction; some gaits with good locomotion performance that were not found by numerical predictions are identified in experiments (e.g., the gaits with $r = 1/7, \Delta\varphi = -1\pi/20$ and $r = 7.3/0.7, \Delta\varphi = 1\pi/10$, illustrated in Figure S11c and Figure S11d, Supporting Information); the absolute errors for the gaits at the boundary of the parameter plane are usually larger than the errors for the gaits locate around the center (see Figure S11h, Supporting Information).

Several factors may account for the discrepancies: (1) The relationship between the resistance force and the segment length in the friction model does not fully match the actual situation; (2) the operation conditions for the forward and backward motions of the robot are not the same due to the hardware limitations and non-uniform mass distribution; (3) due to the limited torque of the servomotor, the deformation profile of the robot segment while dragging multiple segments is not consistent with the measured deformation profile at no load; (4) the robot segments are handmade and therefore may have inconsistencies in shape or size (e.g., the degree of bending of the spring-steel belts and the tightness of the servomotor-driven cords vary in different segments).

Supporting Figures

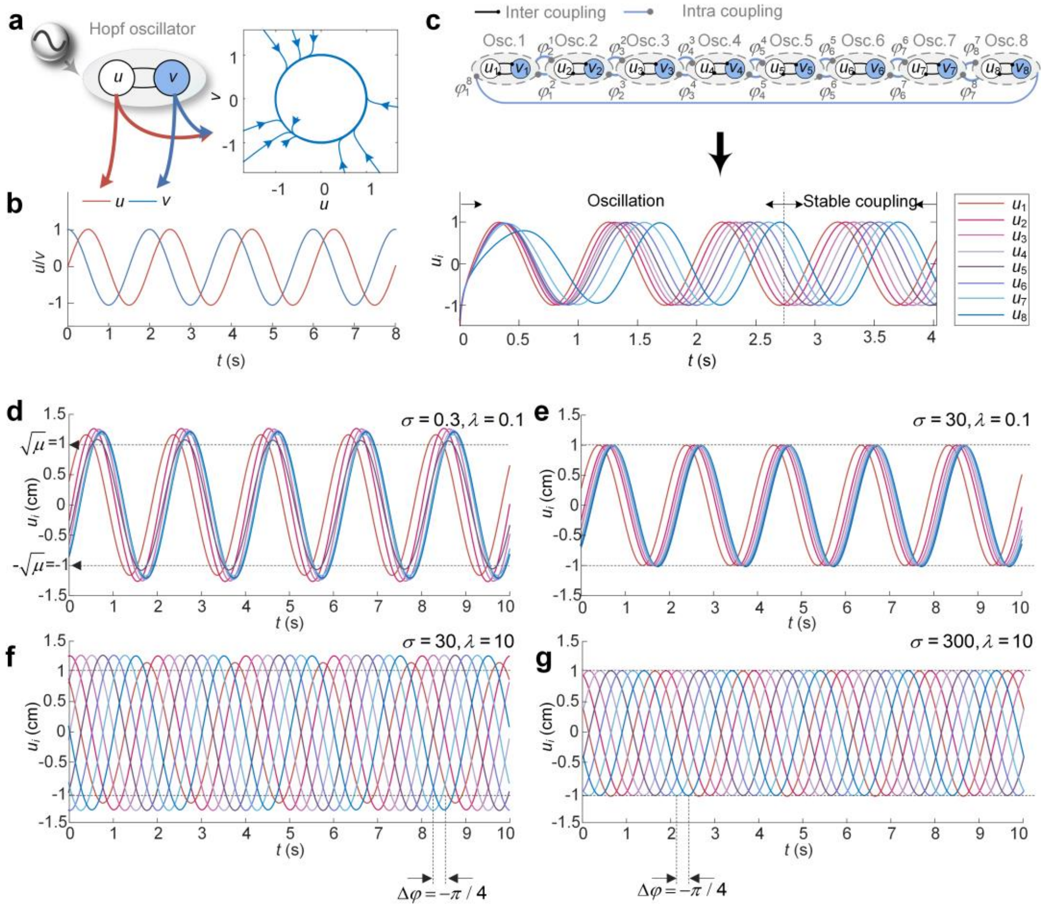

Figure S1. Topological structure and output of the CPG network. a) Structure of the Hopf oscillator and the phase portrait of two state variables u and v. b) Time histories of the two-state variables u and v. c) The CPG-based network consisting of eight Hopf oscillators, which outputs stable sinusoidal signals after the transient stage. The phase difference between all adjacent oscillators is $-3\pi/20$ for bidirectional coupling. d) ~g) Outputs of the CPG-based network with different values of σ and λ, while the values of μ and $\Delta\varphi$ are fixed $\sqrt{\mu}=1$ and $\Delta\varphi=-\pi/4$. d $\sigma=0.3$, $\lambda=0.1$. e $\sigma=30$, $\lambda=0.1$. f $\sigma=30$, $\lambda=10$. g $\sigma=300$, $\lambda=10$.

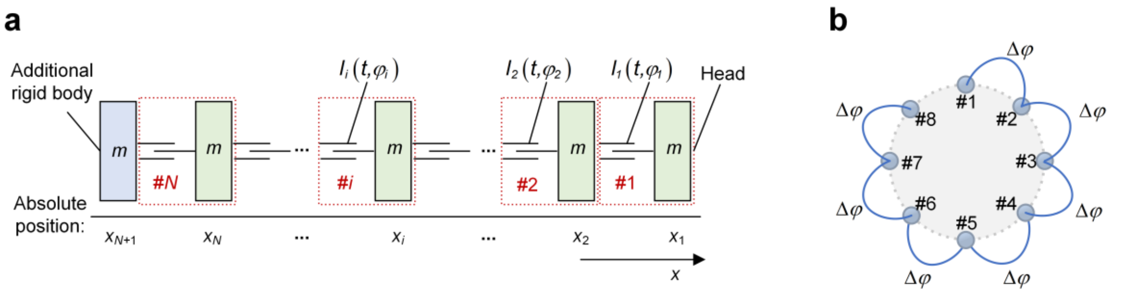

Figure S2. Schematic illustration of the TS phase-coordinated control. a) The model of an *N*-segment metameric robot, where different actuation phases are indicated. b) The IPD pattern of an eight-point system.

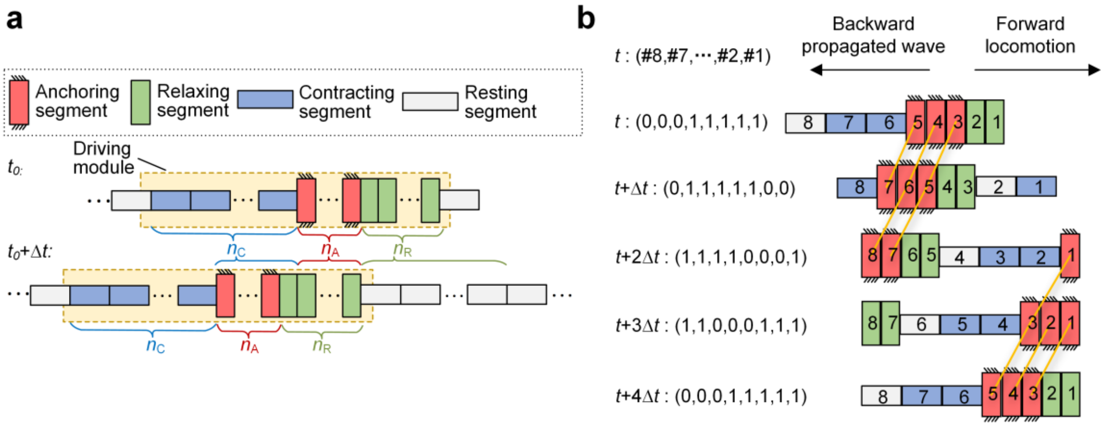

Figure S3. Schematic illustration of the discrete gait control. a) Illustration of a general driving module composed of n_C contracting segments, n_A anchoring segments, and n_R relaxing segments. The driving modules at time instants t_0 and $t_0 + \Delta t$ are indicated by dashed rectangles. The locomotion direction is assumed to be toward the right. b) Schematic illustration of the locomotion of an eight-segment robot under discrete gait G9, where the retrograde peristalsis wave is denoted by yellow inclined lines.

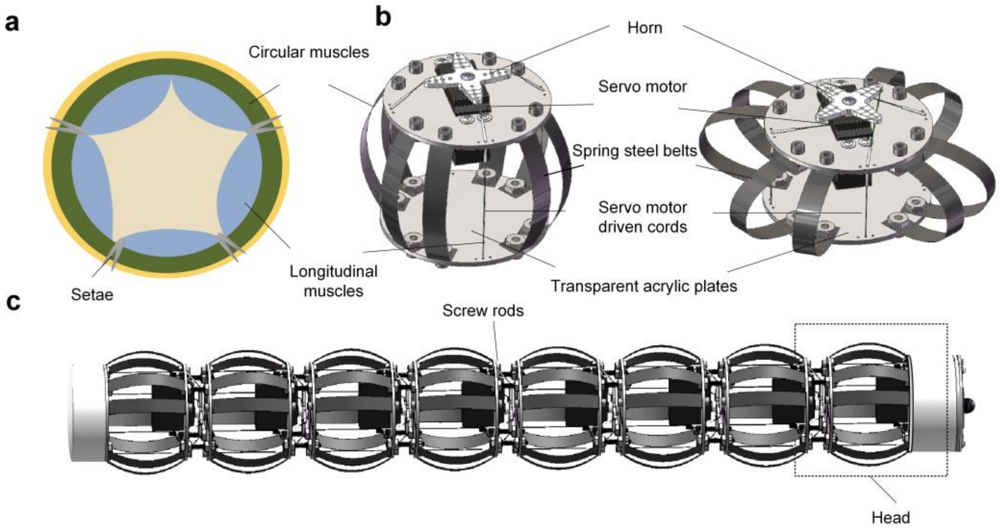

Figure S4. Bioinspired design of the earthworm-like robot. a) Schematic illustration of the cross-section of an earthworm's body. b) Design of the robot segment (Left: fully-relaxed state. Right: fully-contracted state) c) Design of the eight-segment earthworm-like locomotion robot.

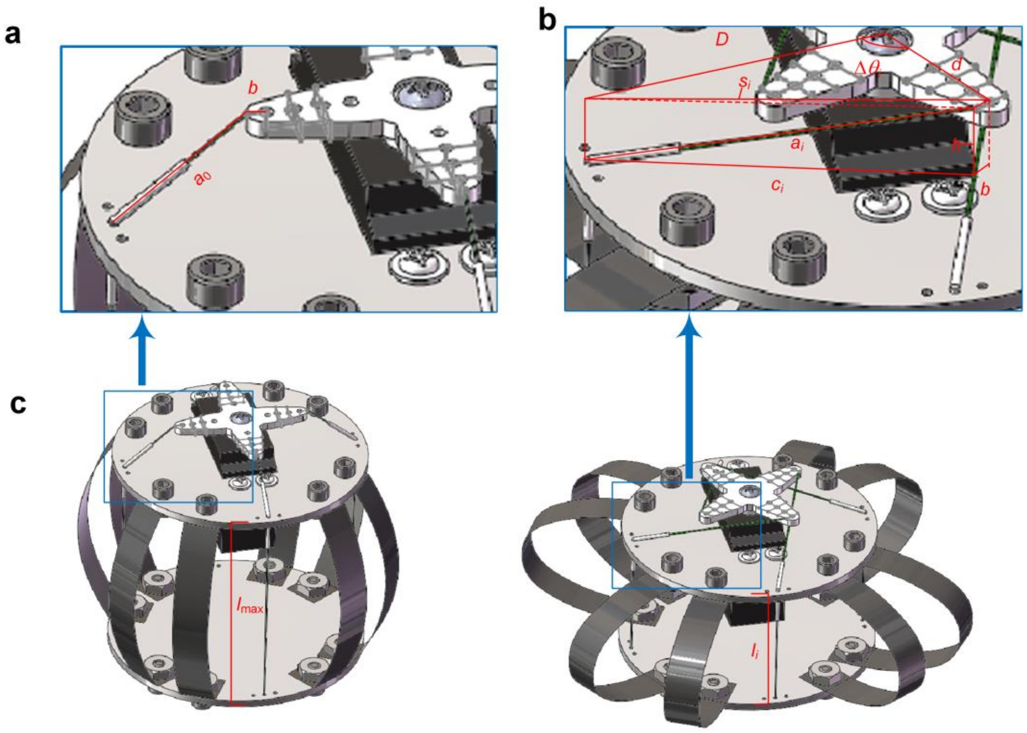

Figure S5. Geometric relations of the robot segment during the rotation of the servomotor. a) Enlarged view of the top of the robot segment in its fully-relaxed state. b Enlarged view of the top of the robot segment when the servomotor rotates $\Delta\theta_i$. c) Comparison of the robot segment before and after axial contraction of Δl_i.

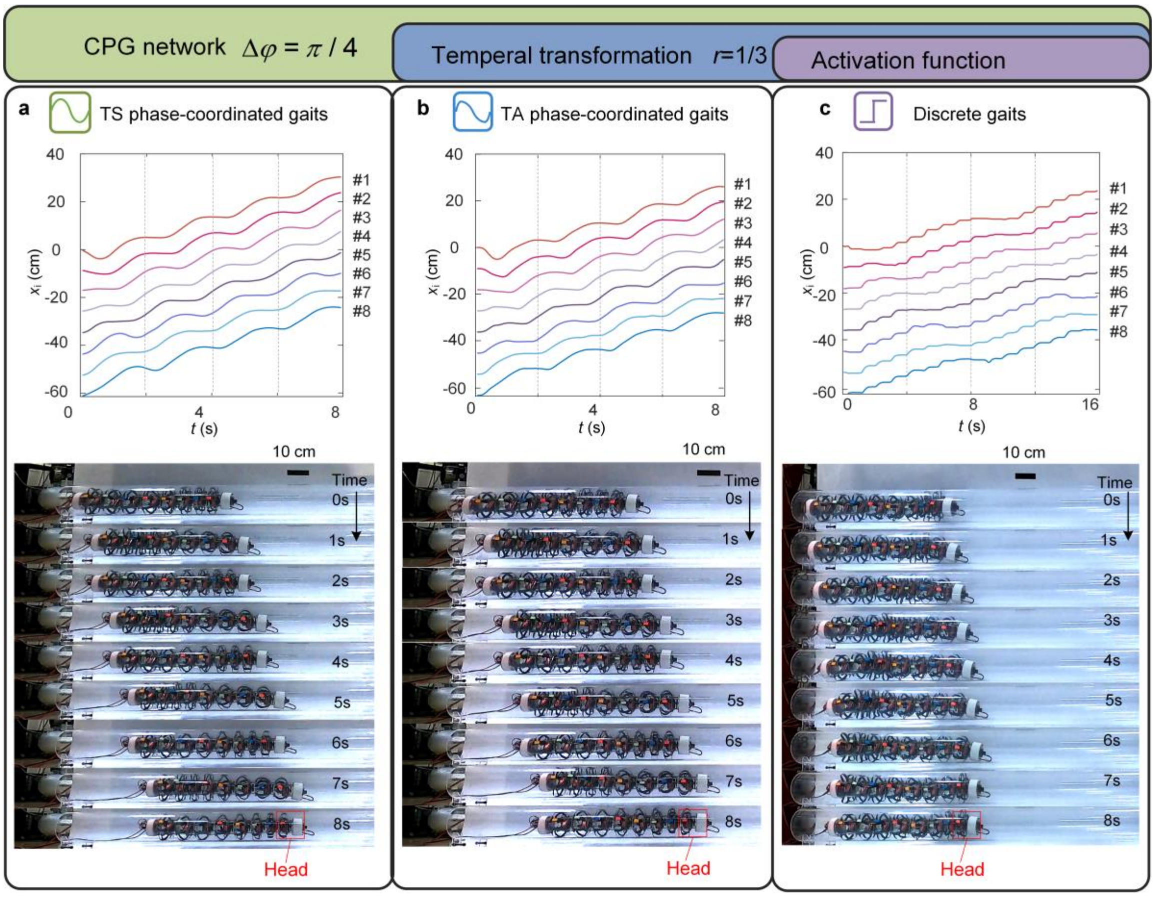

Figure S6. Rectilinear locomotion of the robot controlled by three gaits generated by the CPG-based framework. The displacement-time histories of the eight segments and the snap-shots of the robot during locomotion are demonstrated. a) the TS phase-coordinated gait, b) the TA phase-coordinated gait, and c) the discrete gait.

a

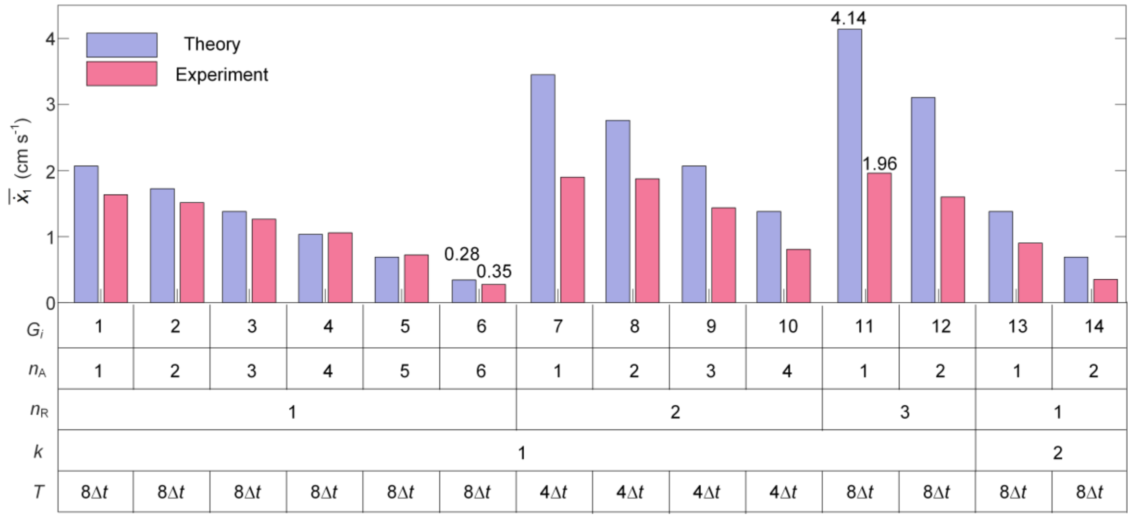

b

<table>
<tr><td>G_i</td><td>1</td><td>2</td><td>3</td><td>4</td><td>5</td><td>6</td><td>7</td><td>8</td><td>9</td><td>10</td><td>11</td><td>12</td><td>13</td><td>14</td></tr>
<tr><td>n_A</td><td>1</td><td>2</td><td>3</td><td>4</td><td>5</td><td>6</td><td>1</td><td>2</td><td>3</td><td>4</td><td>1</td><td>2</td><td>1</td><td>2</td></tr>
<tr><td>n_R</td><td colspan="6">1</td><td colspan="4">2</td><td colspan="2">3</td><td colspan="2">1</td></tr>
<tr><td>k</td><td colspan="12">1</td><td colspan="2">2</td></tr>
<tr><td>T</td><td>$8\Delta t$</td><td>$8\Delta t$</td><td>$8\Delta t$</td><td>$8\Delta t$</td><td>$8\Delta t$</td><td>$8\Delta t$</td><td>$4\Delta t$</td><td>$4\Delta t$</td><td>$4\Delta t$</td><td>$4\Delta t$</td><td>$8\Delta t$</td><td>$8\Delta t$</td><td>$8\Delta t$</td><td>$8\Delta t$</td></tr>
</table>

Figure S7. Locomotion performance under the fourteen admissible discrete gaits of the 8-segment robot. a) Theoretical and experimental average locomotion velocity of the robot under the fourteen discrete gaits. b) The gait parameters and locomotion period corresponding to the fourteen discrete gaits.

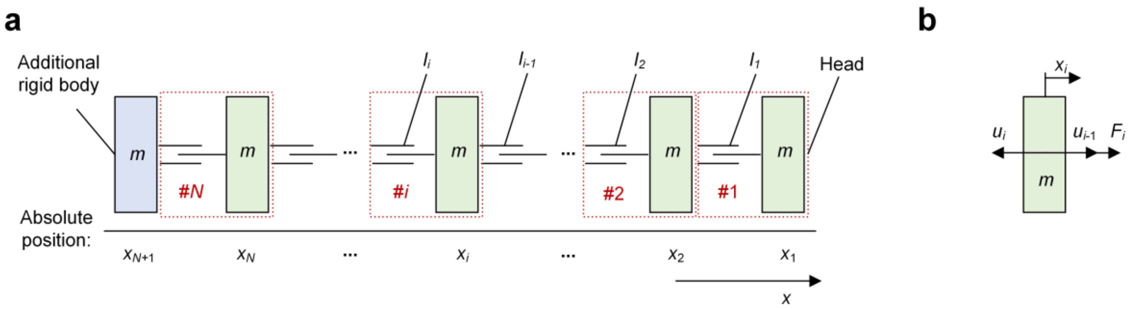

Figure S8. The dynamic model of the earthworm-like robot. a) The dynamic model of an *N*-segment metameric earthworm-like robot. b) Force analysis of a single segment.

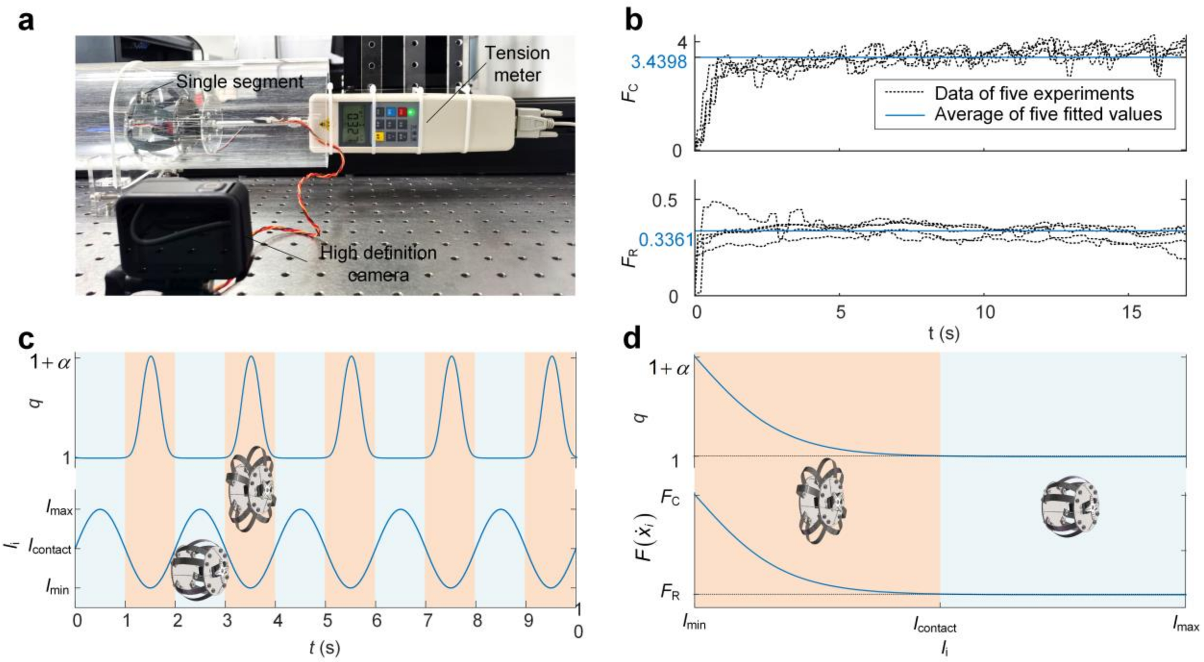

Figure S9. Resistance measurement of the single segment a) The experiment setup. b) Five measurements of F_C and F_R, and the average of the fitted five measurements (truncated steady motion only). c) The relationship between q and l_i. The red part indicates that the segment is in the axial contraction state ($l_i < L_0$), and the blue part indicates the axial expansion state ($l_i > L_0$). d) The relationship between q and l_i, and the relationship between $F(\dot{x}_1)$ and l_i in the proposed resistance model, the red part and the blue part are the same as defined in c.

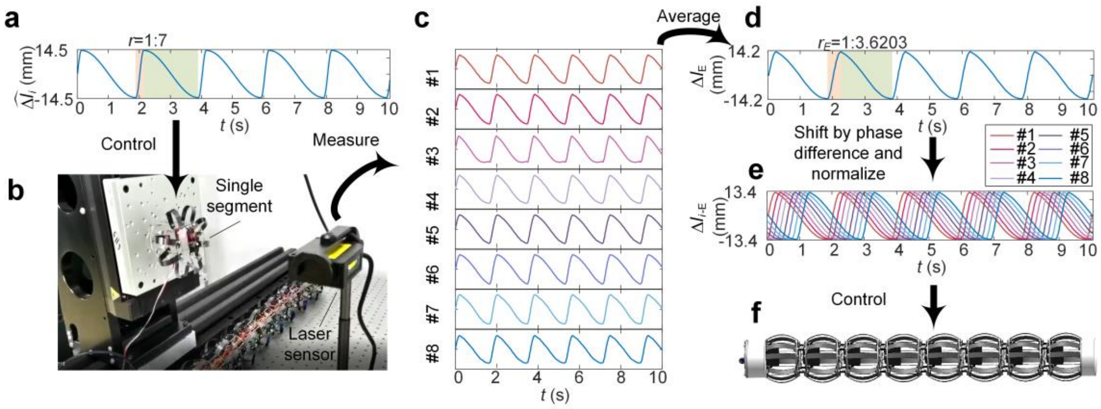

Figure S10. Measurement of the axial deformations of robot segments. a) The desired axial deformation Δl_i derived from u_i of a TA phase-coordinated gait with $r = 1/7$. b) The experimental setup. c) The measured time histories of the axial deformations of the eight robot segments when controlled by the TA phase-coordinated gait with $r = 1/7$. d) The averaged curved of the measured time-histories in c. e) Normalizing Δl_{E} to $\overline{\Delta l_{\mathrm{E}}}$ and shifting Δl_{E} by a prescribed phase difference yields the signals $\Delta l_{i-\mathrm{E}}$ for controlling the robot shown in f.

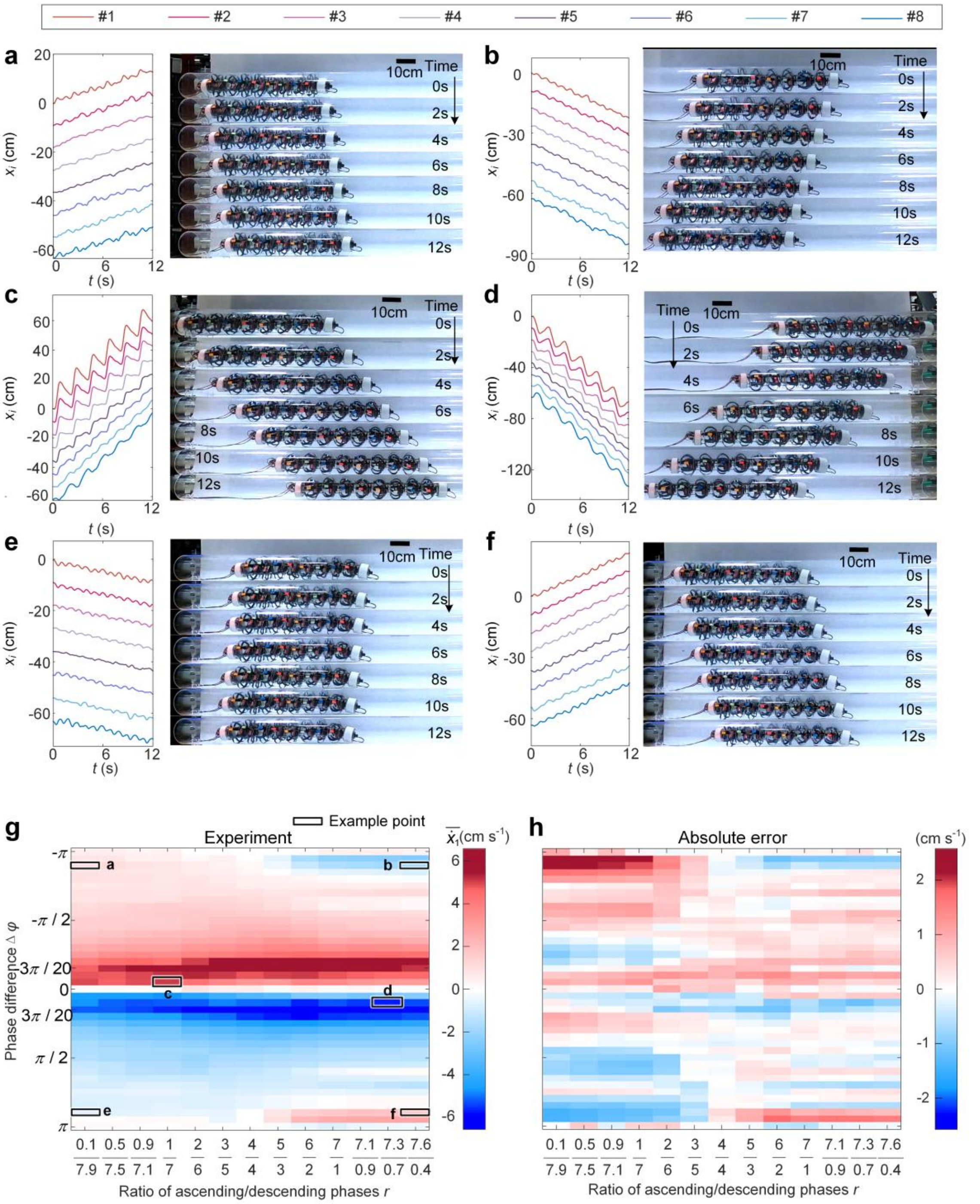

Figure S11. Examples of some TA phase-coordinated gaits. a) -f) The snap-shots of the moving robot and the displacement-time histories of the robot segment corresponding to the exemplified gaits. a). $r = 0.1/7.9$, $\Delta\varphi = -9\pi/10$. b). $r = 7.6/0.4$, $\Delta\varphi = -9\pi/10$. c). $r = 1/7$, $\Delta\varphi = -1\pi/20$. d). $r = 7.3/0.7$, $\Delta\varphi = \pi/10$ e). $r = 0.1/7.9$, $\Delta\varphi = 9\pi/10$. f). $r = 7.6/0.4$, $\Delta\varphi = 9\pi/10$. g). Contour plot of the experimental average steady-state velocity in the $\Delta\varphi - r$ plane, where the exemplified gaits are denoted. h) Errors between the theoretical (Figure 4c) and experimental (Figure 4d) average steady-state velocity.

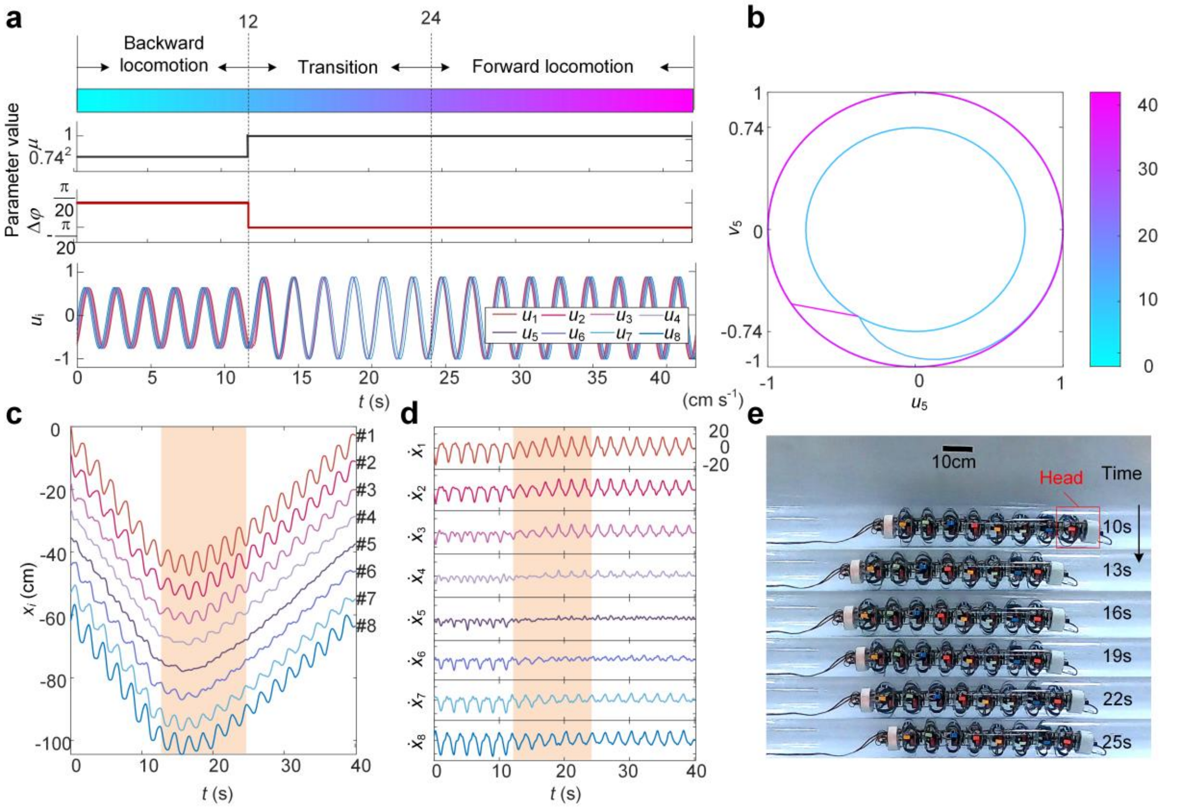

Figure S12. Gait transitions in the gait switching. a) Evolution of the output signals u_i during the transition from a backward locomotion gait ($r = 1$, $\Delta\varphi = \pi / 20$, $\mu = 0.74^2$) to a forward locomotion gait ($r = 1$, $\Delta\varphi = -\pi / 20$, $\mu = 1$). b) Evolution of the limit cycle in the $u_5 - v_5$ plane during the gait switch in a. c), d) The displacement-time histories and the velocity-time histories of the robot segments during the gait switch in a. $\dot{x}_i$ is the velocity of the ith segment. The smooth transition stage is denoted by shades. e) The snap-shots of the moving robot during the gait switching in a from $t = 10$ s to $t = 25$ s.

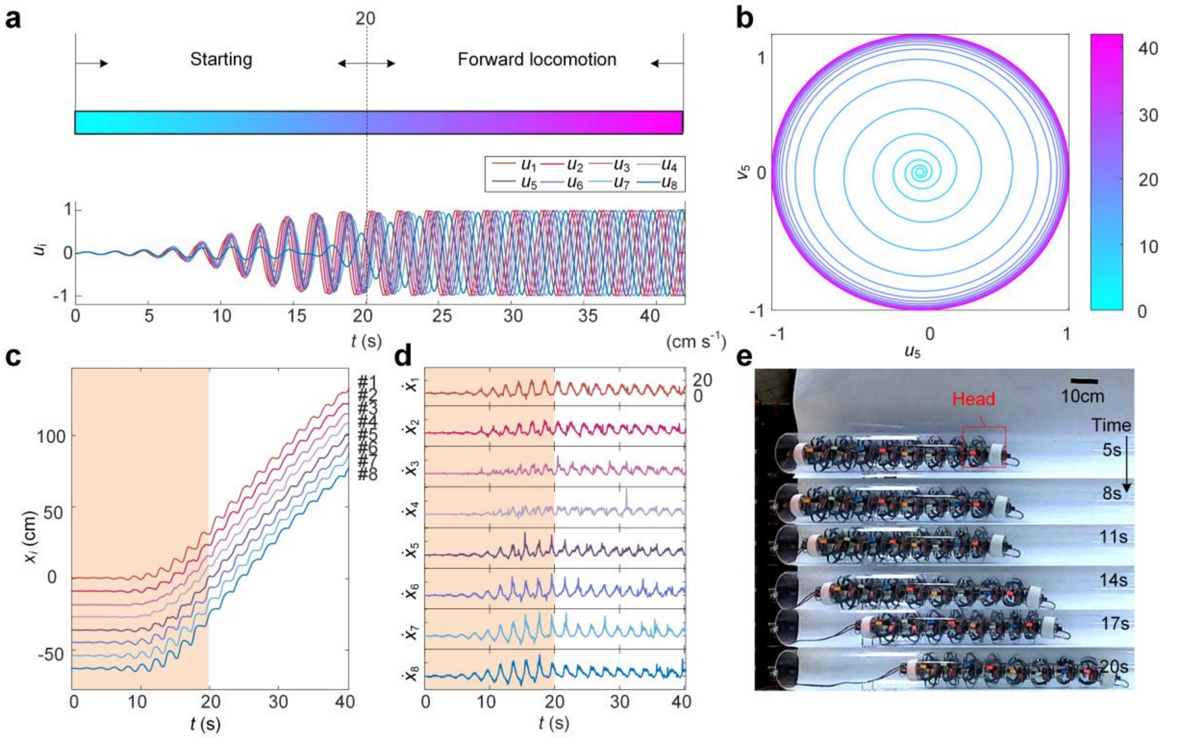

Figure S13. Gait transitions in the starting. a) Evolution of the output signals u_i during the transition from the standstill to a forward locomotion gait ($r=1$, $\Delta\varphi=-\pi/4$, $\mu=1$) without any modulation in parameters involved. b) Evolution of the limit cycle in the u_5-v_5 plane during the starting process in a. c), d) The displacement-time histories and the velocity-time histories of the robot segments during the starting process in a. The smooth starting stage is denoted by shades. e) The snap-shots of the moving robot during the starting process in a from $t=5$ s to $t=20$ s.

Supporting Tables

Table S1. The parameters of the control framework for generating the 14 admissible discrete gaits of an 8-segment robot

Gait	Gait parameters				Parameters of the CPG-based control framework		
	k	n_A	n_R	T	φ_{i+1}^{i} $(i=1,\cdots,7)$ $(\varphi_1^8 = 2\pi - \sum_{i=1}^{7}\varphi_{i+1}^{i})$	r_{ij}^{a}, r_{ij}^{d}	$r_{ij} = r_{ij}^{a} / r_{ij}^{d}$
G1	1	1	1	$8\Delta t$	$\pi/4$	$1/2, 3/2$	$2/6$
G2	1	2	1	$8\Delta t$	$\pi/4$	$3/4, 5/4$	$3/5$
G3	1	3	1	$8\Delta t$	$\pi/4$	$1, 1$	$4/4$
G4	1	4	1	$8\Delta t$	$\pi/4$	$5/4, 3/4$	$5/2$
G5	1	5	1	$8\Delta t$	$\pi/4$	$3/2, 1/2$	$6/2$
G6	1	6	1	$8\Delta t$	$\pi/4$	$7/4, 1/4$	$7/1$
G7	1	1	2	$4\Delta t$	0 (i=1,3,5,7) $\pi/2$ (i=2,4,6)	$1, 1$ (i=1,3,5,7) $1/2, 3/2$ (i=2,4,6,8)	$4/4$ (i=1,3,5,7) $2/6$ (i=2,4,6,8)
G8	1	2	2	$4\Delta t$	$\pi/2$ (i=1,3,5,7) 0 (i=2,4,6)	$1, 1$	$4/4$
G9	1	3	2	$4\Delta t$	0 (i=1,3,5,7) $\pi/2$ (i =2,4,6)	$3/2, 1/2$ (i=1,3,5,7) $1, 1$ (i=2,4,6,8)	$6/2$ (i=1,3,5,7) $4/4$ (i=2,4,6,8)
G10	1	4	2	$4\Delta t$	$\pi/2$ (i =1,3,5,7) 0 (i=2,4,6)	$3/2, 1/2$	$6/2$
G11	1	1	3	$8\Delta t$	$3\pi/4$	$1/2, 1/4$ (j=3k+1) $1/4, 1/2$ (j=3k+2) $1/4, 1/4$ (j=3k+3) $k = 0,1,2,\cdots$	$2/1$ (j=3k+1) $1/2$ (j=3k+2) $1/1$ (j=3k+3)
G12	1	2	3	$8\Delta t$	$3\pi/4$	$1/2, 1/4$ (j=3k+1) $1/2, 1/4$ (j=3k+2) $1/4, 1/4$ (j=3k+3) $k = 0,1,2,\cdots$	$2/1$ (j=3k+1) $2/1$ (j=3k+2) $1/1$ (j=3k+3)
G13	2	1	1	$8\Delta t$	$\pi/4$	$1/2, 3/4$ (j=2k+1) $1/2, 1/4$ (j=2k+2) $k = 0,1,2,\cdots$	$2/3$ (j=2k+1) $2/1$ (j=2k+2)
G14	2	2	1	$8\Delta t$	$\pi/4$	$3/4, 1/4$ (j=2k+1) $3/4, 1/4$ (j=2k+2) $k = 0,1,2,\cdots$.	$3/1$ (j=2k+1) $3/1$ (j=2k+2)

Table S2. Measured ratio r_E and the measured magnitude of Δl_E

**Parameters of Δl_i**		**Parameters of Δl_E**		**Selected for optimization or not**
r	**Magnitude/mm**	r_E	**Magnitude /mm**	
0.1/7.9		1/4.4802	13.42	√
0.2/7.8		1/4.1538	13.52	
0.3/7.7		1/4.1869	13.64	
0.4/7.6		1/4.1832	13.69	
0.5/7.5		1/3.9894	13.84	√
0.6/7.4		1/4.3996	13.97	
0.7/7.3		1/3.9921	14.05	
0.8/7.2		1/3.8341	14.22	
0.9/7.1		1/3.8758	14.30	√
1/7		1/3.6203	14.21	√
2/6		1/2.7432	14.27	√
3/5		1/1.7035	14.31	√
4/4	14.50	1/1.0889	14.42	√
5/3		1.3975/1	14.37	√
6/2		1.8367/1	14.11	√
7/1		2.7221/1	14.14	√
7.1/0.9		3.0644/1	14.03	√
7.2/0.8		2.7206/1	14.06	
7.3/0.7		3.4308/1	13.99	√
7.4/0.6		2.9716/1	13.87	
7.5/0.5		3.1883/1	13.91	
7.6/0.4		3.5167/1	13.78	√
7.7/0.3		3.4730/1	13.83	
7.8/0.2		3.3888/1	13.69	
7.9/0.1		3.2677/1	13.64	

Supporting Movies

Movie S1. Three types of gaits under the same set of parameters

Movie S2. Comparison of three TA phase-coordinated gaits corresponding to different values of r

Movie S3. Gait transition from forward to backward

Movie S4. Gait transition from backward to forward

Movie S5. Gait transition in braking

Movie S6. Gait transition in starting

Supporting References

[1] H. Fang, S. Li, K. W. Wang, J. Xu, *Bioinspiration and Biomimetics* **2015**, *10*, 066006.

[2] H. Fang, S. Li, K. W. Wang, J. Xu, *Multibody Syst. Dyn.* **2015**, *34*, 391.

[3] Z. He, H. Fang, J. Xu, *Jiqiren/Robot* **2020**, *42*, 697.

[4] H. Fang, C. Wang, S. Li, K. W. Wang, J. Xu, *Multibody Syst. Dyn.* **2015**, *35*, 153.
